# One-pot, sequential four-component synthesis of novel heterocyclic [3.3.3] propellane derivatives at room temperature†

**DOI:** 10.1039/c8ra01648h

**Published:** 2018-04-17

**Authors:** Maryam Beyrati, Alireza Hasaninejad

**Affiliations:** Department of Chemistry, Faculty of Sciences, Persian Gulf University Bushehr 7516913817 Iran a_hasaninejad@yahoo.com

## Abstract

An efficient, one-pot, two-step, four-component reaction for the synthesis of propellane derivatives is described by the condensation reaction between acenaphthenequinone, malono derivatives, primary amines and β-ketoester or β-diketone derivatives in the presence of triethylamine in ethanol at room temperature. Using this procedure, all the products were obtained in good to excellent yields.

## Abstract 2

An efficient synthesis of propellane derivatives is described by the condensation reaction between acenaphthenequinone, malono derivatives, primary amines and β-ketoesters or β-diketones in the presence of triethylamine in ethanol at room temperature.

## Graphical abstract

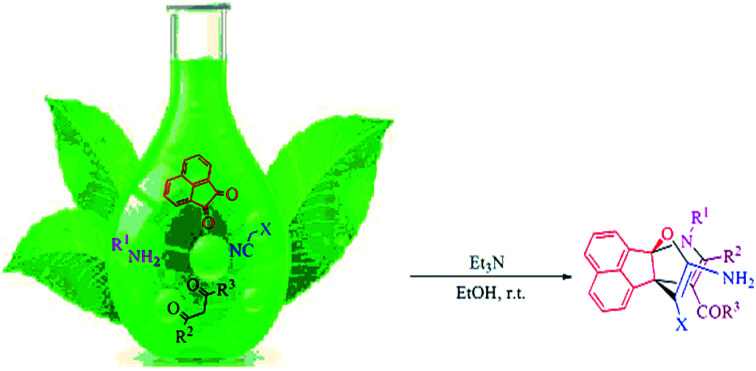

## Introduction

In modern organic chemistry, the improvement of reaction efficiency, the reduction of waste, the avoidance of toxic reagents and the responsible utilization of our resources have become critical objectives.^1,2^ Multi-component reactions, are useful tools for the synthesis of biologically and pharmacologically important compounds because of their environmentally friendly, green characteristics and atom efficient.^3–6^ Therefore, the design and application of multi-component reactions has attracted great attention from research groups working in areas such as biological, medicinal and organic chemistry.

Heterocyclic compounds occur commonly in nature and are essential to life.^7^ These compounds have received considerable attention in recent times due to their wide applications. Functionalized nitrogen and oxygen-containing heterocycles are of importance as they have various industrial, pharmaceutical and biological applications.^8–20^

**Fig. 1:**
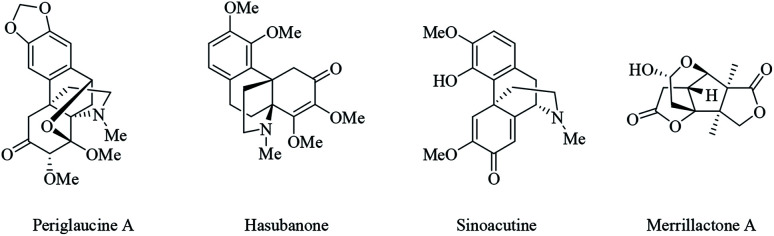
Typical natural and biological products containing propellane skeleton.

Propellanes are tricyclic compounds in which the three rings are connected to a common C–C single bond.^21^ Nitrogen- and oxygen-containing propellanes and their analogues constitute the structural framework of many natural and non-natural products such as periglaucine A, hasubanone, sinoacutine and merrilactone A (Fig. 1).^22,23^ Propellanes due to their challenging framework and broad spectra of biological and pharmacological activities, constitute an interesting class of compounds in organic chemistry.^24^

**Scheme 1:**
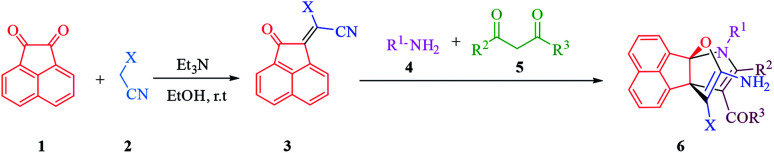
The synthesis of propellane derivatives *via* the reaction between acenaphthoquinone, malono derivatives, dicarbonyl compounds and primary amines in the presence of Et_3_N in EtOH at room temperature.

Recently, there have been some reports about the synthesis of propellane compounds from ninhydrin^25–32^ and acenaphthoquinone.^32–34^ In continuation of our research on the synthesis of biologically important heterocycles and green chemistry protocols,^35–47^ we report a simple and efficient method for the synthesis of novel propellane derivatives *via* a one-pot, sequential four-component condensation reaction between acenaphthoquinone (1), malono derivatives (2), primary amines (4) and β-ketoester or β-diketone (5) derivatives in the presence of triethylamine in ethanol at room temperature (Scheme 1).

## Results and discussion

In the first step, the reaction between acenaphthoquinone (1), malononitrile (2a), ethyl acetoacetate (5a) and benzyl amine (4c), as a model reaction, was investigated in the presence of different molar ratios of Et_3_N in some solvents (5 mL) at room temperature and the results are summarized in Table 1. For this propose to a stirred mixture of acenaphthoquinone 1 (1 mmol) and malononitrile 2a (1 mmol) in EtOH (5 mL) at room temperature different amount of Et_3_N were added to form the corresponding Knoevenagel adduct and then ethyl acetoacetate 5a (1 mmol) and benzyl amine 4c (1 mmol) were added to obtain the corresponding product 6a. As it is shown in Table 1, higher yield and shorter reaction time were obtained when the reaction was carried out in the presence of 1 mmol of the Et_3_N in ethanol at room temperature (Table 1, entry 4). It should be mention that simultaneity of this four-component condensation reaction was studied in the presence of 1 mmol Et_3_N in EtOH at room temperature and we observed that benzyl amine has remained intact in this condition and the corresponding propellane was not formed.

**Table 1:** Optimization of the reaction conditions^a^

Entry	Amount of Et_3_N (mmol)	Solvent	Time (h)	Yield^b^ (%)
1	—	EtOH	5	—
2	0.1	EtOH	5	45
3	0.5	EtOH	5	87
4	1.0	EtOH	1.2	91
5	1.5	EtOH	1.2	91
6	1.0	H_2_O	2.0	70
7	1.0	DMF	4.0	60
8	1.0	MeOH	1.5	85
9	1.0	CHCl_3_	4.0	40
10	1.0	THF	4.0	55
11	1.0	CH_3_CN	40	45

a Triethylamine (1.0 mmol) was added to a stirred mixture of acenaphthoquinone 1 (1 mmol), malononitrile 2a (1 mmol) in EtOH (5 mL) at room temperature and after about 10 min to form the corresponding Knoevenagel adduct, then ethyl acetoacetate 5a (1 mmol) and benzyl amine 4c (1 mmol) were added to obtain the corresponding product 6a.

b Isolated yields.

**Fig. 2:**
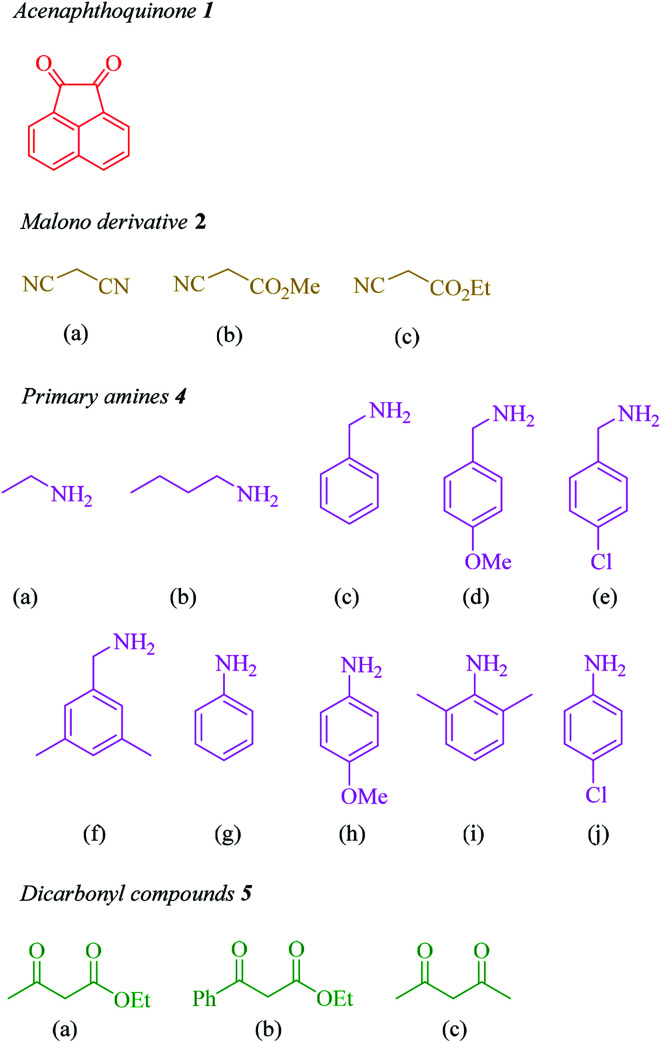
Diversity elements employed for synthesis of propellane derivatives.

In the next step, the scope and efficiency of the process was explored under the optimized conditions. For this purpose, acenaphthoquinone was condensed with alkyl malonates, aliphatic and aromatic amines and dicarbonyl compounds in the presence of Et_3_N (1 mmol) to afford the corresponding products 6. The structural diversity of reactants is summarized in Fig. 2 and the results are displayed in Table 2.

**Table 2:** One-pot, sequential four component synthesis of propellane derivatives in the presence of Et_3_N (1 mmol) in EtOH at room temperature.^a^^,^^b^

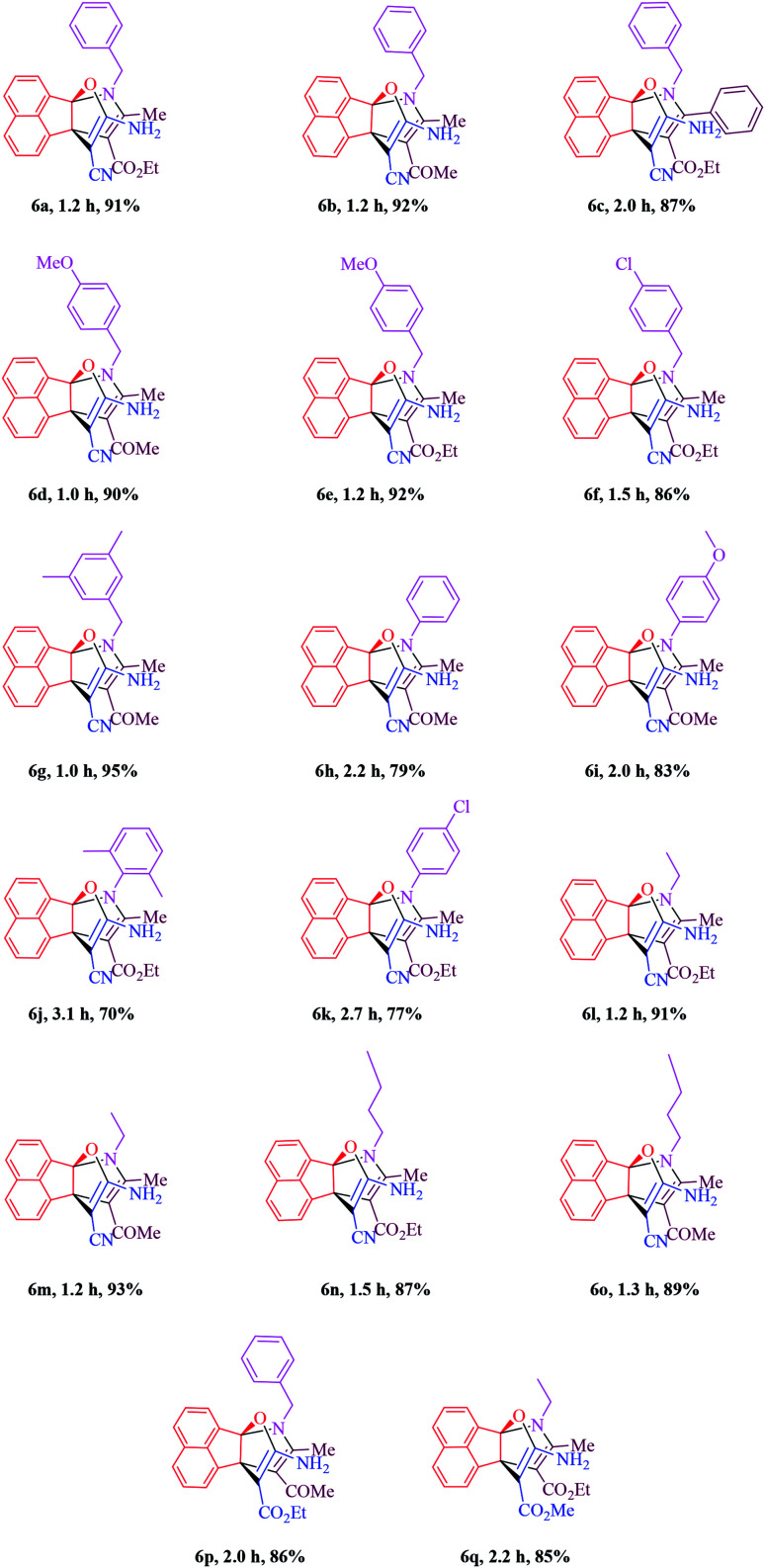

a Triethylamine (1.0 mmol) was added to a stirred mixture of acenaphthoquinone 1 (1 mmol), malono derivatives 2 (1 mmol) in EtOH (5 mL) at room temperature to form the corresponding Knoevenagel adduct (about 10–30 min), then dicarbonyl compound 5 (1 mmol) and primary amine 4 (1 mmol) were added to obtain the desired product 6.

b Isolated yields.

The synthetic pathway for the synthesis of titled compounds is consisting of two steps. At first, compound 3 are obtained from acenaphthoquinone 1 and alkyl malonates 2. Then, the resulting products are treated with primary amines 4 and β-dicarbonyl compounds 5 to afford the related propellane derivatives as the desired products. As Table 2 indicates, a variety of alkyl malonates, aliphatic and aromatic amines and β-dicarbonyl compounds were successfully applied in this process to afford the corresponding propellane derivatives as novel compounds in good to excellent yields.

To demonstrate the generality and scope of this method, malono derivatives, various acyclic β-dicarbonyl compounds such as methyl acetoacetate, ethyl acetoacetate and ethyl benzoylacetate were treated with a broad range of aliphatic and aromatic amines in the presence of Et_3_N (1 mmol) in EtOH at room temperature and the results are shown in Table 2. Use of malononitrile leads to shorter reaction times and higher yields compared to application of other alkylmalonates such as methyl cyanoacetate or ethyl cyanoacetate (compare 6b with 6p and 6l with 6q). As it is shown in Table 2, benzyl amines bearing electron-donating or -withdrawing substituents gave the desired propellanes in high yields and in short reaction times and it seems that when the methoxy group is placed on the aromatic ring, reactivity is better than when the chlorine group is on the ring. Moreover, the presence of 3,5-dimethyl group on the aromatic ring has the best efficiency. Some aromatic amines were also screened to carry out the four-component reaction by this method and the results are listed in Table 2 (entries 6h–6j). Generally, aliphatic amines are more reactive than aromatic amines. Ortho-substituted anilines, whatever the nature of the substituted groups, required a longer reaction period. For instance, reaction of acenaphthenequinone, malononitrile and 2,6-dimethylaniline with ethyl acetoacetate gave the desired product (6j) in 70% yield after 3.1 h. Moreover, aliphatic amines such as ethyl amine and butyl amine were used for the synthesis of the corresponding product in excellent yields (Table 2, entries 6l–6o). In the same way, Some cyclic and acyclic 1.3-dicarbonyl compounds were also screened to carry out the one pot four-component reaction by this method and the results are listed in Table 2. As it is shown in Table 2, aliphatic β-keto esters are more reactive than aromatic β-keto esters (compare 6a and 6c) and no products were obtained when cyclic 1.3-dicarbonyl compounds were involved in this one-pot room temperature reaction.

**Scheme 2:**
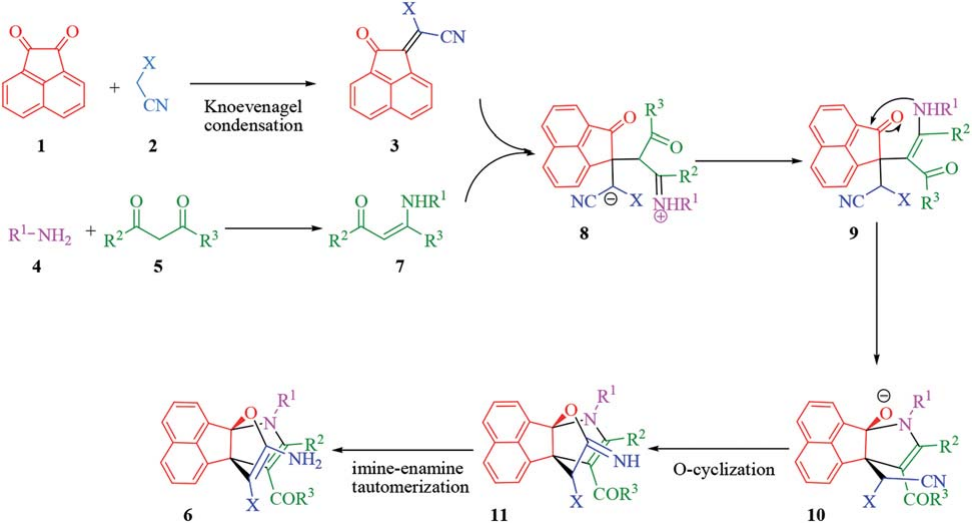
The proposed mechanism for the synthesis of propellane 6 in the presence of Et_3_N.

The proposed mechanism for the synthesis of propellane compounds 6 in the presence of Et_3_N is shown in Scheme 2. Based on this mechanism, Et_3_N is effective catalyst for the formation of olefin (3) which readily prepares *in situ* by Knoevenagel condensation of acenaphthoquinone (1) with the active methylene of compound (2). Next, the formation of enamine (7) occurs through condensation of amine (4) with dicarbonyl compound (5). Then, the enamine (7) attacks to olefin (3) in a Michael addition to produce intermediate (8). After proton transfer and tautomerization, intermediate (8) converts to intermediate (10), through intramolecular cyclization by nitrogen attack of enamine to carbonyl group. In this stage, nucleophilic addition of amino group to C=O bond afforded intermediate (10). Then, intramolecular *O*-cyclization and the tautomerization of imino group to amino group lead to propellanes (6).

## Experimental

All chemicals were purchased from Merck or Fluka chemical companies. The ^1^H NMR (400 MHz) and ^13^C NMR (100 MHz) were run on a Bruker Avance 400. Melting points were recorded on a Stuart Scientific Apparatus SMP3 (UK) in open capillary tubes. Elemental C, H and N analyses were performed using a Costech CHNS-O elemental analyzer.

### General procedure for the synthesis of propellane derivatives 6

Triethylamine (1.0 mmol) was added to a stirred mixture of acenaphthoquinone 1 (1 mmol), malono derivatives 2 (1 mmol) in EtOH (5 mL) and the reaction mixture was stirred to complete the formation of related Knoevenagel adduct 3 (monitored by TLC). Subsequently, dicarbonyl compound 5 (1 mmol) and primary amine 4 (1 mmol) were added to this reaction mixture at once and dropwise over 10 min, respectively and reacted at room temperature for the appropriate amount of time (see Table 2). After completion of the reaction confirmed by TLC, the reaction mixture was filtered and washed with ethanol to afford the pure product 6a–l.

#### Ethyl-11-amino-7-benzyl-10-cyano-8-methyl-7*H*-6*b*,9*a*-(epoxyetheno)acenaphtho[1,2-*b*]pyrrole-9-carboxylate 6a

White powder, mp = 249–251 °C, ^1^H NMR (DMSO-*d*_6_, 400 MHz) *δ* (ppm): 1.45 (t, *J* = 6.6 Hz, 3H), 2.25 (s, 3H), 4.04 (q, *J* = 7.2 Hz, 2H), 4.54 (d, *J* = 16.0 Hz, 1H), 4.60 (d, *J* = 16.0 Hz, 1H), 7.57 (s, 2H), 7.66–7.71 (m, 3H), 7.73–7.81 (m, 4H), 7.89 (d, *J* = 8.0 Hz, 2H), 8.04 (d, *J* = 7.2 Hz, 2H). ^13^C NMR (DMSO-*d*_6_, 100 MHz) *δ* (ppm): 14.4, 14.6, 58.1, 60.5, 61.2, 70.7, 79.9, 110.4, 118.4, 120.6, 121.0, 124.7, 125.8, 127.8, 129.2, 129.6, 132.0, 135.8, 136.3, 142.7, 147.2, 147.6, 147.7, 162.4, 166.5, 166.9, 168.0, 168.1. Anal. calcd for C_28_H_23_N_3_O_3_: C, 74.82; H, 5.16; N, 9.35%. Found: C, 74.85; H, 5.14; N, 9.33%. IR (KBr) (*ν*_max_, cm^−1^): 3358 and 3192 (NH_2_), 2193 (CN), 1687 (C=O), 1652 (OC=C).

#### 9-Acetyl-11-amino-7-benzyl-8-methyl-7*H*-6*b*,9*a*-(epoxyetheno)acenaphtho[1,2-*b*]pyrrole-10-carbonitrile 6b

White powder, mp = 250–252 °C, ^1^H NMR (DMSO-*d*_6_, 400 MHz) *δ* (ppm): 2.32 (s, 3H), 2.36 (s, 3H), 4.74 (d, *J* = 15.6 Hz, 1H), 4.99 (d, *J* = 15.6 Hz, 1H), 7.45 (s, 2H), 7.63 (t, *J* = 7.4 Hz, 2H), 7.69–7.71 (m, 2H), 7.73–7.77 (m, 3H), 7.85 (d, *J* = 7.6 Hz, 2H), 8.02 (d, *J* = 8.0 Hz, 2H). ^13^C NMR (DMSO-*d*_6_, 100 MHz) *δ* (ppm): 15.9, 30.3, 58.4, 59.1, 72.2, 79.8, 116.4, 116.6, 118.5, 120.4, 121.4, 121.6, 124.4, 124.9, 127.7, 128.9, 129.5, 131.9, 135.9, 136.3, 143.0, 148.0, 156.6, 157.1, 166.5, 167.2, 192.7. Anal. calcd for C_27_H_21_N_3_O_2_: C, 77.31; H, 5.05; N, 10.02%. Found: C, 77.32; H, 5.02; N, 10.05%.

#### Ethyl-11-amino-7-benzyl-10-cyano-8-phenyl-7*H*-6*b*,9*a*-(epoxyetheno)acenaphtho[1,2-*b*]pyrrole-9-carboxylate 6c

White powder, mp = 247–249 °C, ^1^H NMR (DMSO-*d*_6_, 400 MHz) *δ* (ppm): 1.38 (t, *J* = 7.0 Hz, 3H), (q, *J* = 6.8 Hz, 2H), 4.92 (d, *J* = 16.0 Hz, 1H), 4.98 (d, *J* = 16.0 Hz, 1H), 7.43 (t, *J* = 7.4 Hz, 3H), 7.51 (t, *J* = 7.0 Hz, 3H), 7.66 (s, 2H), 7.70–7.77 (m, 4H), 7.83 (d, *J* = 6.8 Hz, 1H), 7.87 (d, *J* = 7.2 Hz, 1H), 7.93 (d, *J* = 8.0 Hz, 2H), 8.07 (d, *J* = 8.0 Hz, 2H). ^13^C NMR (DMSO-*d*_6_, 100 MHz) *δ* (ppm): 15.3, 43.6, 47.1, 47.2, 72.2, 79.9, 113.0, 116.3, 117.3, 117.5, 121.2, 123.4, 125.4, 125.7, 127.0, 127.7, 127.9, 128.3, 130.3, 134.5, 134.7, 135.6, 139.3, 147.4, 152.6, 155.4, 158.5, 158.5, 159.1, 159.3, 162.2, 168.1. Anal. calcd for C_33_H_25_N_3_O_3_: C, 77.48; H, 4.93; N, 8.21%. Found: C, 77.51; H, 4.91; N, 8.19%.

#### 9-Acetyl-11-amino-7-(4-methoxybenzyl)-8-methyl-7*H*-6*b*,9*a*-(epoxyetheno)acenaphtho[1,2-*b*]pyrrole-10-carbonitrile 6d

White powder, mp = 249–251 °C, ^1^H NMR (DMSO-*d*_6_, 400 MHz) *δ* (ppm): 2.32 (s, 3H), 2.37 (s, 3H), 3.75 (s, 3H), 4.67 (d, *J* = 16.0 Hz, 1H), 4.78 (d, *J* = 16.0 Hz, 1H), 7.35 (d, *J* = 6.8 Hz, 2H), 7.55–7.59 (m, 2H), 7.77 (t, *J* = 7.6 Hz, 2H), 7.84–7.89 (m, 2H), 8.09 (s, 2H), 8.17 (d, *J* = 7.6 Hz, 2H). ^13^C NMR (DMSO-*d*_6_, 100 MHz) *δ* (ppm): 15.9, 30.3, 57.6, 58.0, 58.4, 72.2, 79.9, 118.5, 120.4, 121.4, 121.7, 124.5, 124.9, 127.7, 128.9, 129.5, 131.9, 135.9, 136.3, 140.0, 140.1, 140.6, 140.8, 143.8, 166.6, 167.2, 168.6, 192.7. Anal. calcd for C_28_H_23_N_3_O_3_: C, 74.82; H, 5.16; N, 9.35%. Found: C, 74.79; H, 5.17; N, 9.38%. IR (KBr) (*ν*_max_, cm^−1^): 3361 and 3101 (NH_2_), 2195 (CN), 1711 (C=O), 1644 (OC=C).

#### Ethyl-11-amino-10-cyano-7-(4-methoxybenzyl)-8-methyl-7*H*-6*b*,9*a*-(epoxyetheno)acenaphtho[1,2-*b*]pyrrole-9-carboxylate 6e

White powder, mp = 251–253 °C, ^1^H NMR (DMSO-*d*_6_, 400 MHz) *δ* (ppm): 0.57 (t, *J* = 7.6 Hz, 3H), 1.90 (s, 3H), 3.10 (s, 3H), 3.45–3.49 (m, 1H), 3.79–3.82 (m, 1H), 4.77 (d, *J* = 16.0 Hz, 1H), 5.03 (d, *J* = 15.6 Hz, 1H), 7.57 (s, 2H), 7.66–7.71 (m, 2H), 7.73–7.76 (m, 2H), 7.79 (t, *J* = 6.0 Hz, 2H), 7.88 (d, *J* = 8.0 Hz, 2H), 8.03 (d, *J* = 7.6 Hz, 2H). ^13^C NMR (DMSO-*d*_6_, 100 MHz) *δ* (ppm): 14.4, 14.6, 47.5, 69.4, 70.0, 73.1, 73.3, 79.7, 106.1, 116.2, 117.6, 121.2, 125.1, 127.0, 127.7, 127.9, 128.2, 130.2, 134.7, 135.5, 139.3, 147.4, 158.7, 159.3, 159.8, 160.7, 162.3, 165.0, 168.8. Anal. calcd for C_29_H_25_N_3_O_4_: C, 72.64; H, 5.25; N, 8.76%. Found: C, 72.62; H, 5.23; N, 8.79%.

#### Ethyl-11-amino-7-(4-chlorobenzyl)-10-cyano-8-methyl-7*H*-6*b*,9*a*-(epoxyetheno)acenaphtho[1,2-*b*]pyrrole-9-carboxylate 6f

White powder, mp = 249–251 °C, ^1^H NMR (DMSO-*d*_6_, 400 MHz) *δ* (ppm): 1.44 (t, *J* = 7.0 Hz, 3H), 2.25 (s, 3H), 4.11–4.17 (m, 2H), 4.42 (d, *J* = 16.0 Hz, 1H), 4.48 (d, *J* = 16.0 Hz, 1H), 7.77 (s, 2H), 7.85–7.91 (m, 2H), 7.92–7.96 (m, 3H), 7.99 (t, *J* = 6.0 Hz, 1H), 8.08 (d, *J* = 8.4 Hz, 2H), 8.23 (d, *J* = 8.0 Hz, 2H). ^13^C NMR (DMSO-*d*_6_, 100 MHz) *δ* (ppm): 14.4, 14.6, 58.1, 60.6, 60.6, 70.7, 79.9, 110.4, 118.4, 120.6, 121.0, 124.7, 125.8, 127.8, 129.1, 129.6, 132.0, 135.8, 136.3, 142.7, 154.8, 155.1, 155.8, 157.5, 157.8, 166.6, 167.2, 167.3. Anal. calcd for C_28_H_22_ClN_3_O_3_: C, 69.49; H, 4.58; Cl, 7.33; N, 8.68%. Found: C, 69.50; H, 4.56; N, 8.70%.

#### 9-Acetyl-11-amino-7-(3,5-dimethylbenzyl)-8-methyl-7*H*-6*b*,9*a*-(epoxyetheno)acenaphtho[1,2-*b*]pyrrole-10-carbonitrile 6g

White powder, mp = 251–253 °C, ^1^H NMR (DMSO-*d*_6_, 400 MHz) *δ* (ppm): 2.05 (s, 3H), 2.10 (s, 3H), 2.31 (s, 3H), 2.36 (s, 3H), 4.96 (d, *J* = 16.0 Hz, 1H), 5.02 (d, *J* = 16.0 Hz, 1H), 6.83 (d, *J* = 7.6 Hz, 1H), 7.02 (t, *J* = 7.4 Hz, 1H), 7.21–7.35 (m, 2H), 7.49 (d, *J* = 7.2 Hz, 2H), 7.58 (d, *J* = 9.2 Hz, 1H), 7.79 (s, 2H), 7.85 (dd, *J* = 2.4 Hz, 8.8 Hz, 1H), 8.02 (d, *J* = 2.4 Hz, 1H). ^13^C NMR (DMSO-*d*_6_, 100 MHz) *δ* (ppm): 14.1, 20.2, 20.5, 30.3, 47.4, 59.3, 71.9, 79.7, 103.9, 108.4, 112.9, 116.9, 122.4, 123.4, 123.6, 125.4, 127.7, 128.4, 128.7, 134.1, 134.5, 137.2, 145.4, 152.4, 154.5, 158.4, 159.2, 167.5, 198.2. Anal. calcd for C_29_H_25_N_3_O_2_: C, 77.83; H, 5.63; N, 9.39%. Found: C, 77.80; H, 5.65; N, 9.41%.

#### 9-Acetyl-11-amino-8-methyl-7-phenyl-7*H*-6*b*,9*a*-(epoxyetheno)acenaphtho[1,2-*b*]pyrrole-10-carbonitrile 6h

White powder, mp = 247–249 °C, ^1^H NMR (DMSO-*d*_6_, 400 MHz) *δ* (ppm): 2.31 (s, 3H), 2.36 (s, 3H), 7.51–7.60 (m, 3H), 7.78–7.82 (m, 2H), 7.88 (s, 2H), 7.97 (dd, *J* = 1.4 Hz, 7.8 Hz, 2H), 8.23 (dd, *J* = 2.2 Hz, 8.6 Hz, 2H), 8.38 (d, *J* = 2.4 Hz, 2H). ^13^C NMR (DMSO-*d*_6_, 100 MHz) *δ* (ppm): 15.8, 30.2, 57.2, 71.4, 79.8, 101.7, 109.6, 112.9, 117.2, 117.5, 123.2, 123.4, 124.6, 125.5, 127.6, 127.8, 128.9, 129.5, 132.8, 134.3, 136.4, 143.2, 152.6, 155.8, 158.9, 199.0. Anal. calcd for C_26_H_19_N_3_O_2_: C, 77.02; H, 4.72; N, 10.36%. Found: C, 77.04; H, 4.75; N, 10.32%.

#### 9-Acetyl-11-amino-7-(4-methoxyphenyl)-8-methyl-7*H*-6*b*,9*a*-(epoxyetheno)acenaphtho[1,2-*b*]pyrrole-10-carbonitrile 6i

White powder, mp = 252–254 °C, ^1^H NMR (DMSO-*d*_6_, 400 MHz) *δ* (ppm): 2.22 (s, 3H), 2.93 (s, 3H), 3.74 (s, 3H), 7.53 (d, *J* = 6.8 Hz, 2H), 7.67 (t, *J* = 7.6 Hz, 1H), 7.89 (t, *J* = 7.4 Hz, 1H), 7.99–8.04 (m, 4H), 8.08 (s, 2H), 8.33 8.02 (d, *J* = 8.4 Hz, 2H). ^13^C NMR (DMSO-*d*_6_, 100 MHz) *δ* (ppm): 15.4, 30.0, 48.1, 57.5, 72.1, 79.2, 101.9, 110.0, 112.9, 117.1, 117.5, 122.5, 123.1, 124.6, 125.5, 129.4, 133.5, 134.2, 142.7, 152.5, 155.6, 158.8, 158.9, 166.1, 167.6, 177.6, 195.0. Anal. calcd for C_27_H_21_N_3_O_3_: C, 74.47; H, 4.86; N, 9.65%. Found: C, 74.43; H, 4.89; N, 9.67%.

#### Ethyl-11-amino-10-cyano-7-(2,6-dimethylphenyl)-8-methyl-7*H*-6*b*,9*a*-(epoxyetheno)acenaphtho[1,2-*b*]pyrrole-9-carboxylate 6j

White powder, mp = 255–257 °C, ^1^H NMR (DMSO-*d*_6_, 400 MHz) *δ* (ppm): 1.35 (t, *J* = 7.2 Hz, 3H), 2.21 (s, 3H), 2.30 (s, 3H), 2.33 (s, 3H), 3.31–3.34 (m, 2H), 7.35 (d, *J* = 6.8 Hz, 2H), 7.55–7.59 (m, 2H), 7.77 (t, *J* = 7.6 Hz, 2H), 7.84–7.89 (m, 2H), 8.10 (s, 2H), 8.17 (d, *J* = 7.6 Hz, 1H). ^13^C NMR (DMSO-*d*_6_, 100 MHz) *δ* (ppm): 13.6, 14.6, 21.4, 21.7, 59.6, 60.2, 72.0, 79.8, 104.3, 108.9, 112.8, 112.9, 116.8, 117.0, 121.5, 123.7, 124.1, 125.3, 125.6, 128.4, 133.9, 134.3, 135.2, 144.6, 152.3, 154.2, 167.6, 170.9, 179.4. Anal. calcd for C_29_H_25_N_3_O_3_: C, 75.14; H, 5.44; N, 9.07%. Found: C, 75.10; H, 5.42; N, 9.12%.

#### Ethyl-11-amino-7-(4-chlorophenyl)-10-cyano-8-methyl-7*H*-6*b*,9*a*-(epoxyetheno)acenaphtho[1,2-*b*]pyrrole-9-carboxylate 6k

White powder, mp = 251–253 °C, ^1^H NMR (DMSO-*d*_6_, 400 MHz) *δ* (ppm): 1.19 (t, *J* = 7.2 Hz, 3H), 2.00 (s, 3H), 4.04 (q, *J* = 7.2 Hz, 2H), 7.56 (d, *J* = 8.8 Hz, 1H), 7.65 (d, *J* = 7.2 Hz, 1H), 7.72 (t, *J* = 7.6 Hz, 1H), 7.77 (s, 2H), 7.84 (dd, *J* = 2.4 Hz, 8.8 Hz, 1H), 7.90 (t, *J* = 7.6 Hz, 1H), 8.03–8.06 (m, 3H), 8.37 (d, *J* = 8.0 Hz, 2H). ^13^C NMR (DMSO-*d*_6_, 100 MHz) *δ* (ppm): 13.7, 14.6, 59.8, 60.2, 71.9, 79.2, 103.0, 108.9, 113.1, 116.9, 119.8, 123.5, 125.3, 126.1, 134.1, 136.2, 142.4, 151.2, 152.4, 155.1, 158.7, 159.3, 167.2, 167.7, 180.2. Anal. calcd for C_27_H_20_ClN_3_O_3_: C, 69.01; H, 4.29; Cl, 7.54; N, 8.94%. Found: C, 69.05; H, 4.26; N, 8.92%.

#### Ethyl-11-amino-10-cyano-7-ethyl-8-methyl-7*H*-6*b*,9*a*-(epoxyetheno)acenaphtho[1,2-*b*]pyrrole-9-carboxylate 6l

White powder, mp = 250–252 °C, ^1^H NMR (DMSO-*d*_6_, 400 MHz) *δ* (ppm): 0.76 (t, *J* = 7.2 Hz, 3H), 1.44 (t, *J* = 7.0 Hz, 3H), 2.25 (s, 3H), 2.81 (q, *J* = 7.0 Hz, 2H), 4.30 (q, *J* = 7.0 Hz, 2H), 7.57 (s, 2H), 7.66–7.71 (m, 1H), 7.72–7.76 (m, 2H), 7.79 (t, *J* = 6.0 Hz, 1H), 7.88 (d, *J* = 8.4 Hz, 1H), 8.03 (d, *J* = 8.0 Hz, 1H). ^13^C NMR (DMSO-*d*_6_, 100 MHz) *δ* (ppm): 7.9, 14.4, 14.6, 58.1, 58.6, 60.5, 70.7, 79.9, 110.4, 118.4, 120.6, 121.0, 124.7, 125.8, 127.8, 129.1, 129.6, 132.0, 135.8, 136.3, 142.7, 167.3, 168.1. Anal. calcd for C_23_H_21_N_3_O_3_: C, 71.30; H, 5.46; N, 10.85%. Found: C, 71.28; H, 5.44; N, 10.88%. IR (KBr) (*ν*_max_, cm^−1^): 3405 and 3278 (NH_2_), 2197 (CN), 1663 (C=O), 1639 (OC=C).

#### 9-Acetyl-11-amino-7-ethyl-8-methyl-7*H*-6*b*,9*a*-(epoxyetheno)acenaphtho[1,2-*b*]pyrrole-10-carbonitrile 6m

White powder, mp = 251–253 °C, ^1^H NMR (DMSO-*d*_6_, 400 MHz) *δ* (ppm): 0.86 (t, *J* = 7.0 Hz, 3H), 2.32 (s, 3H), 2.36 (s, 3H), 3.81 (q, *J* = 7.1 Hz, 2H), 7.46 (s, 2H), 7.63 (t, *J* = 7.6 Hz, 1H), 7.70 (d, *J* = 7.2 Hz, 1H), 7.75 (t, *J* = 7.0 Hz, 1H), 7.85 (d, *J* = 7.6 Hz, 2H), 8.01 (d, *J* = 7.6 Hz, 1H). ^13^C NMR (DMSO-*d*_6_, 100 MHz) *δ* (ppm): 15.3, 15.9, 30.3, 58.4, 59.0, 72.2, 79.9, 118.5, 120.4, 121.4, 121.6, 124.4, 124.9, 127.7, 128.9, 129.5, 131.9, 135.9, 136.3, 143.0, 167.2, 192.7. Anal. calcd for C_22_H_19_N_3_O_2_: C, 73.93; H, 5.36; N, 11.76%. Found: C, 73.92; H, 5.39; N, 11.73%.

#### Ethyl-11-amino-7-butyl-10-cyano-8-methyl-7*H*-6*b*,9*a*-(epoxyetheno)acenaphtho[1,2-*b*]pyrrole-9-carboxylate 6n

White powder, mp = 247–249 °C, ^1^H NMR (DMSO-*d*_6_, 400 MHz) *δ* (ppm): 0.95 (t, *J* = 7.2 Hz, 3H), 1.44 (t, *J* = 7.2 Hz, 3H), 1.80–1.92 (m, 4H), 2.25 (s, 3H), 2.98 (t, *J* = 6.4 Hz, 2H), 4.32 (q, *J* = 7.2 Hz, 2H), 7.57 (s, 2H), 7.67 (t, *J* = 7.6 Hz, 1H), 7.71 (d, *J* = 7.6 Hz, 1H), 7.75 (d, *J* = 6.4 Hz, 1H), 7.79 (t, *J* = 6.4 Hz, 1H), 7.88 (d, *J* = 8.0 Hz, 1H), 8.03 (d, *J* = 8.0 Hz, 1H). ^13^C NMR (DMSO-*d*_6_, 100 MHz) *δ* (ppm): 12.7, 14.4, 14.6, 19.0, 26.2, 55.1, 58.1, 60.5, 70.7, 79.9, 110.4, 118.4, 120.6, 121.0, 124.7, 125.8, 127.8, 129.1, 129.6, 132.0, 135.8, 136.3, 142.7, 167.3, 168.1. Anal. calcd for C_25_H_25_N_3_O_3_: C, 72.27; H, 6.06; N, 10.11%. Found: C, 72.30; H, 6.05; N, 10.08%. IR (KBr) (*ν*_max_, cm^−1^): 3389 and 3179 (NH_2_), 2203 (CN), 1661 (C=O), 1635 (OC=C).

#### 9-Acetyl-11-amino-7-butyl-8-methyl-7*H*-6*b*,9*a*-(epoxyetheno)acenaphtho[1,2-*b*]pyrrole-10-carbonitrile 6o

White powder, mp = 248–250 °C, ^1^H NMR (DMSO-*d*_6_, 400 MHz) *δ* (ppm): 0.84 (t, *J* = 7.2 Hz, 3H), 1.05–1.12 (m, 4H), 2.31 (s, 3H), 2.36 (s, 3H), 2.96 (q, *J* = 7.3 Hz, 2H), 7.46 (s, 2H), 7.63 (t, *J* = 7.6 Hz, 1H), 7.70 (d, *J* = 7.2 Hz, 1H), 7.75 (t, *J* = 7.0 Hz, 1H), 7.85 (d, *J* = 7.2 Hz, 2H), 8.01 (d, *J* = 8.0 Hz, 1H). ^13^C NMR (DMSO-*d*_6_, 100 MHz) *δ* (ppm): 15.2, 15.9, 23.7, 29.9, 30.3, 58.4, 59.5, 72.2, 79.9, 118.5, 120.4, 121.4, 121.6, 124.4, 124.9, 127.7, 128.9, 129.5, 131.9, 135.9, 136.3, 166.6, 167.2, 192.7. Anal. calcd for C_24_H_23_N_3_O_2_: C, 74.78; H, 6.01; N, 10.90%. Found: C, 74.75; H, 6.03; N, 10.92%.

#### Ethyl-9-acetyl-11-amino-7-benzyl-8-methyl-7*H*-6*b*,9*a*-(epoxyetheno)acenaphtho[1,2-*b*]pyrrole-10-carboxylate 6p

White powder, mp = 249–251 °C, ^1^H NMR (DMSO-*d*_6_, 400 MHz) *δ* (ppm): 0.83 (t, *J* = 7.0 Hz, 3H), 2.10 (s, 3H), 2.44 (s, 3H), 3.80 (q, *J* = 7.3 Hz, 2H), 4.84 (d, *J* = 16.0 Hz, 1H), 5.12 (d, *J* = 16.0 Hz, 1H), 7.42 (s, 2H), 7.64–7.67 (m, 2H), 7.70–7.74 (m, 3H), 7.84 (d, *J* = 6.4 Hz, 3H), 7.99–8.06 (m, 3H). ^13^C NMR (DMSO-*d*_6_, 100 MHz) *δ* (ppm): 15.0, 15.1, 31.9, 58.7, 59.4, 71.8, 72.5, 80.0, 118.2, 118.4, 120.1, 120.8, 124.0, 124.1, 125.6, 127.5, 128.6, 129.1, 131.8, 136.2, 136.6, 142.9, 163.4, 166.1, 166.7, 167.3, 167.9, 195.3. Anal. calcd for C_29_H_26_N_2_O_4_: C, 74.66; H, 5.62; N, 6.00%. Found: C, 74.68; H, 5.59; N, 6.02%. IR (KBr) (*ν*_max_, cm^−1^): 3359 and 3161 (NH_2_), 1738 (C=O), 1667 (C=O), 1639 (OC=C).

#### 9-Ethyl-10-methyl-11-amino-7-ethyl-8-methyl-7*H*-6*b*,9*a*-(epoxyetheno)acenaphtho[1,2-*b*]pyrrole-9,10-dicarboxylate 6q

White powder, mp = 247–249 °C, ^1^H NMR (DMSO-*d*_6_, 400 MHz) *δ* (ppm): 0.86 (t, *J* = 7.0 Hz, 3H), 1.18 (t, *J* = 7.2 Hz, 3H), 2.00 (s, 3H), 3.22 (s, 3H), 3.81 (q, *J* = 7.1 Hz, 2H), 4.04 (q, *J* = 7.2 Hz, 2H), 7.38 (s, 2H), 7.59 (t, *J* = 7.6 Hz, 1H), 7.63 (d, *J* = 7.2 Hz, 1H), 7.69 (t, *J* = 6.6 Hz, 1H), 7.81 (d, *J* = 8.4 Hz, 1H), 7.99 (d, *J* = 7.6 Hz, 1H), 8.08 (d, *J* = 6.8 Hz, 1H). ^13^C NMR (DMSO-*d*_6_, 100 MHz) *δ* (ppm): 14.5, 14.6, 15.1, 58.6, 60.1, 60.9, 61.7, 70.8, 79.8, 120.1, 124.0, 124.2, 126.0, 127.6, 128.5, 129.0, 131.7, 136.1, 136.6, 142.7, 164.6, 165.0, 166.2, 166.9. Anal. calcd for C_24_H_24_N_2_O_5_: C, 68.56; H, 5.75; N, 6.66%. Found: C, 68.58; H, 5.77; N, 6.63%. IR (KBr) (*ν*_max_, cm^−1^): 3412 and 3249 (NH_2_), 1736 (C=O), 1675 (C=O), 1642 (OC=C).

## Conclusions

In conclusion, we have reported a highly efficient method for the synthesis of biologically important propellane derivatives *via* a one-pot, sequential four-component condensation reaction presence of triethylamine in ethanol at room temperature. High atom-economy, simple procedure in the excellent yields, easy workup procedure, mild reaction conditions and metal-catalyst free are the main advantages of this method.

## Conflicts of interest

There are no conflicts to declare.

## Supplementary Material

RA-008-C8RA01648H-s001
