# Supplementary material for: One-pot, sequential four-component synthesis of novel heterocyclic [3.3.3] propellane derivatives at room temperature
Source: RSC Adv. 2018 Apr 17;8(26):14171–6. doi: 10.1039/c8ra01648h (PMC9079859; doi:10.1039/c8ra01648h)
Supplement: RA-008-C8RA01648H-s001 [file RA-008-C8RA01648H-s001.pdf]

Electronic Supplementary Material (ESI) for RSC Advances.
This journal is © The Royal Society of Chemistry 2018

One-pot, sequential four-component synthesis of novel heterocyclic [3.3.3] propellane derivatives at room temperature

SUPPORTING INFORMATION

Ethyl-11-amino-7-benzyl-10-cyano-8-methyl-7H-6b,9a-(epoxyetheno)acenaphtho[1,2-b]pyrrole-9-carboxylate (6a)

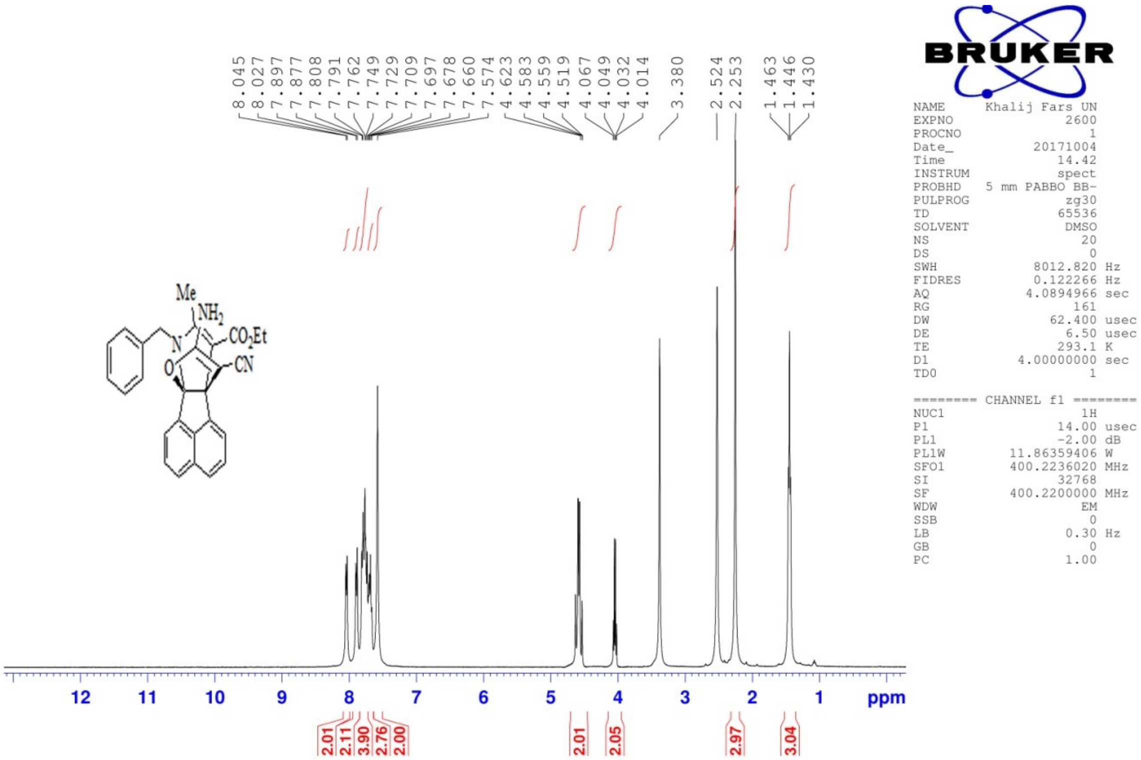

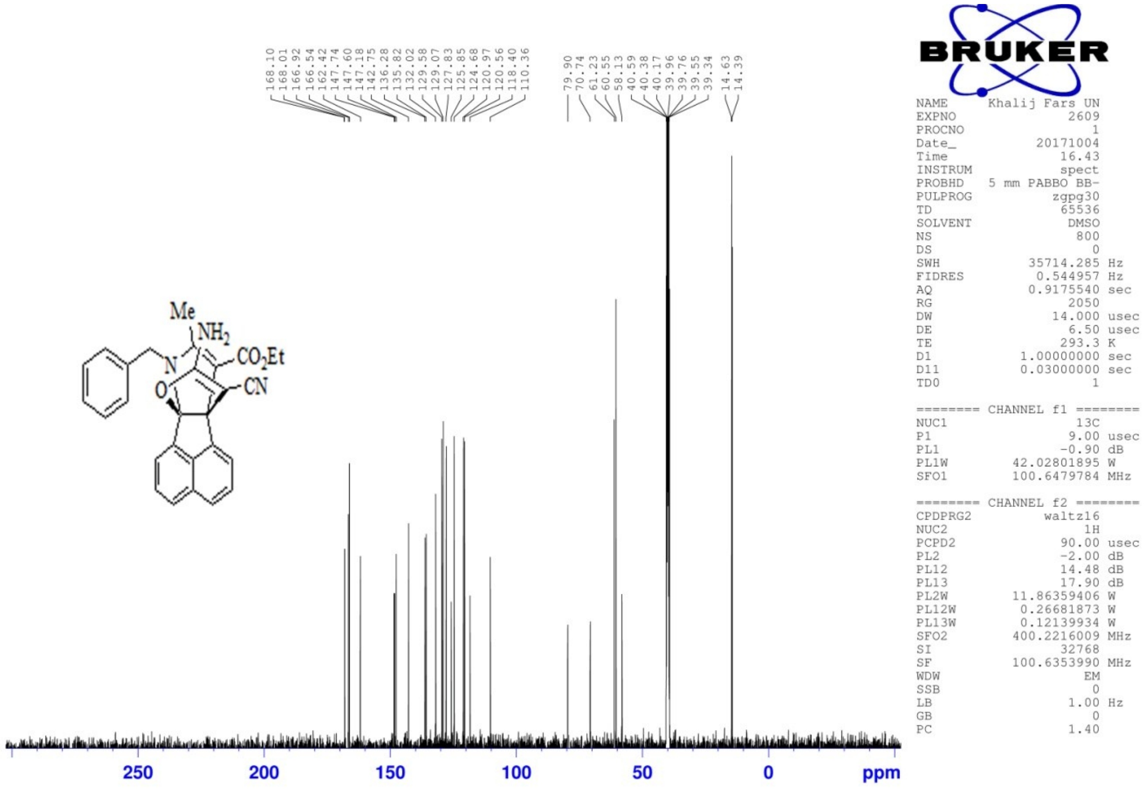
168.10
168.01
166.92
166.54
162.42
147.74
147.60
147.18
142.75
136.28
135.82
132.02
129.58
129.07
127.83
125.85
124.68
120.97
120.56
118.40
110.36
79.90
70.74
61.23
60.55
58.13
40.59
40.38
40.17
39.96
39.76
39.55
39.34
14.63
14.39
Me
NH_2
CO_2Et
N
O
CN
BRUKER
NAME Khalij Fars UN
EXPNO 2609
PROCNO 1
Date_ 20171004
Time 16.43
INSTRUM spect
PROBHD 5 mm PABBO BB-
PULPROG zgpg30
TD 65536
SOLVENT DMSO
NS 800
DS 0
SWH 35714.285 Hz
FIDRES 0.544957 Hz
AQ 0.9175540 sec
RG 2050
DW 14.000 usec
DE 6.50 usec
TE 293.3 K
D1 1.00000000 sec
D11 0.03000000 sec
TD0 1
======== CHANNEL f1 ========
NUC1 13C
P1 9.00 usec
PL1 -0.90 dB
PL1W 42.02801895 W
SFO1 100.6479784 MHz
======== CHANNEL f2 ========
CPDPRG2 waltz16
NUC2 1H
PCPD2 90.00 usec
PL2 -2.00 dB
PL12 14.48 dB
PL13 17.90 dB
PL2W 11.86359406 W
PL12W 0.26681873 W
PL13W 0.12139934 W
SFO2 400.2216009 MHz
SI 32768
SF 100.6353990 MHz
WDW EM
SSB 0
LB 1.00 Hz
GB 0
PC 1.40
250
200
150
100
50
0
ppm

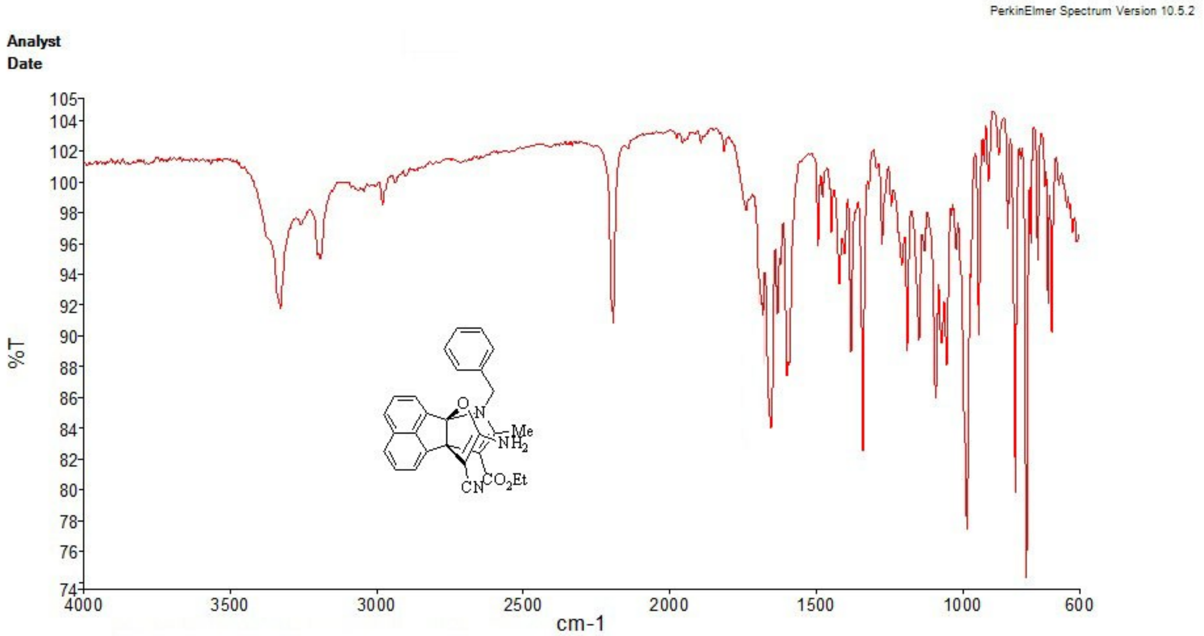
PerkinElmer Spectrum Version 10.5.2
Analyst
Date
%T
105
104
102
100
98
96
94
92
90
88
86
84
82
80
78
76
74
4000
3500
3000
2500
2000
1500
1000
600
cm-1
N
O
Me
NH_2
CN
CO_2Et

9-acetyl-11-amino-7-benzyl-8-methyl-7H-6b,9a-(epoxyetheno)acenaphtho[1,2-b]pyrrole-10-carbonitrile (6b)

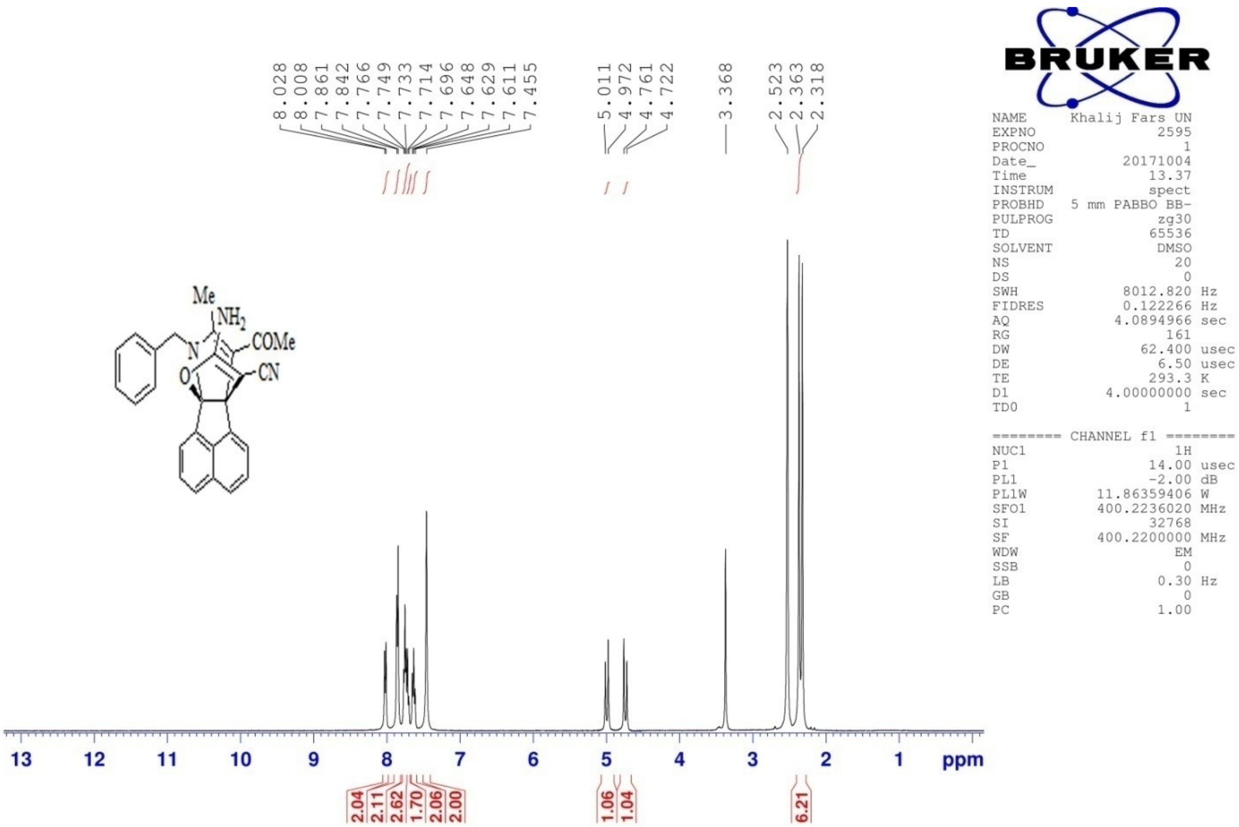

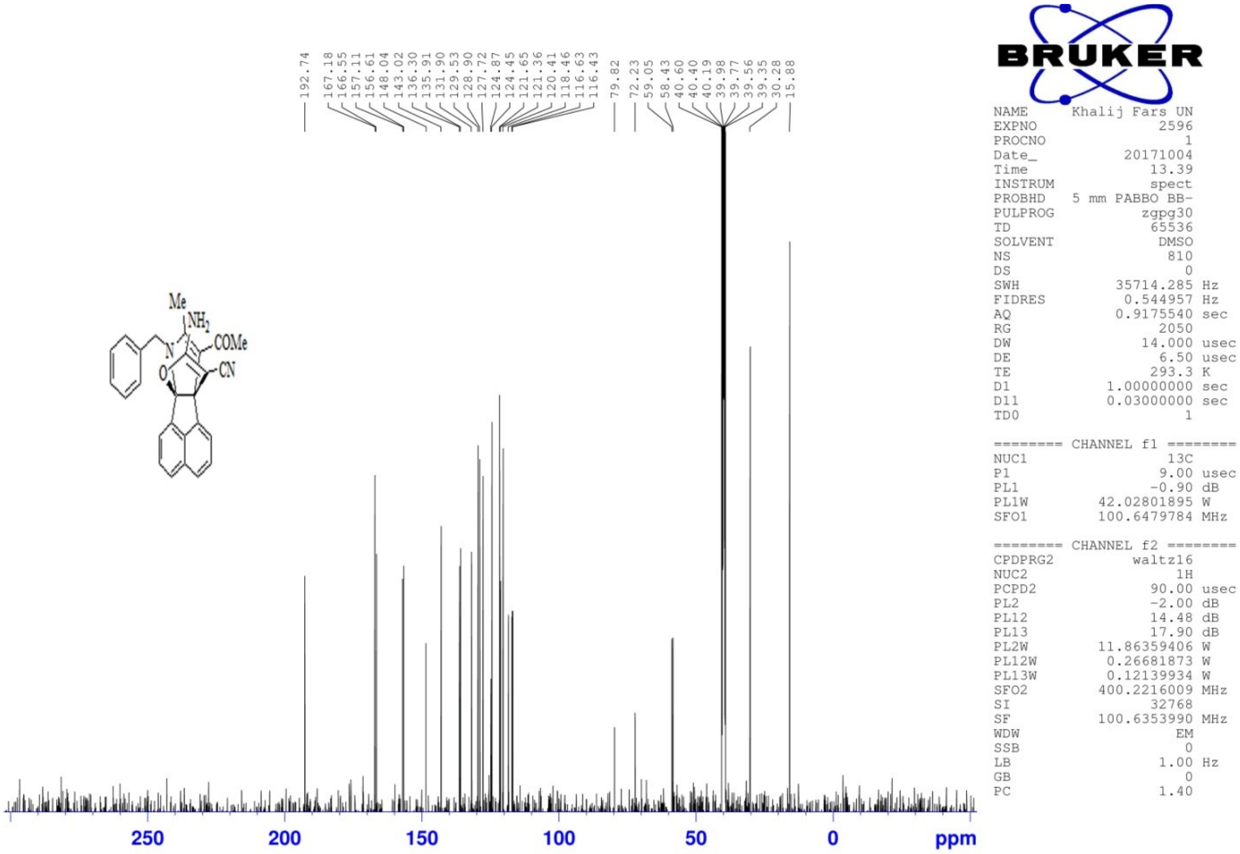

Ethyl-11-amino-7-benzyl-10-cyano-8-phenyl-7H-6b,9a-(epoxyetheno)acenaphtho[1,2-b]pyrrole-9-carboxylate (6c)

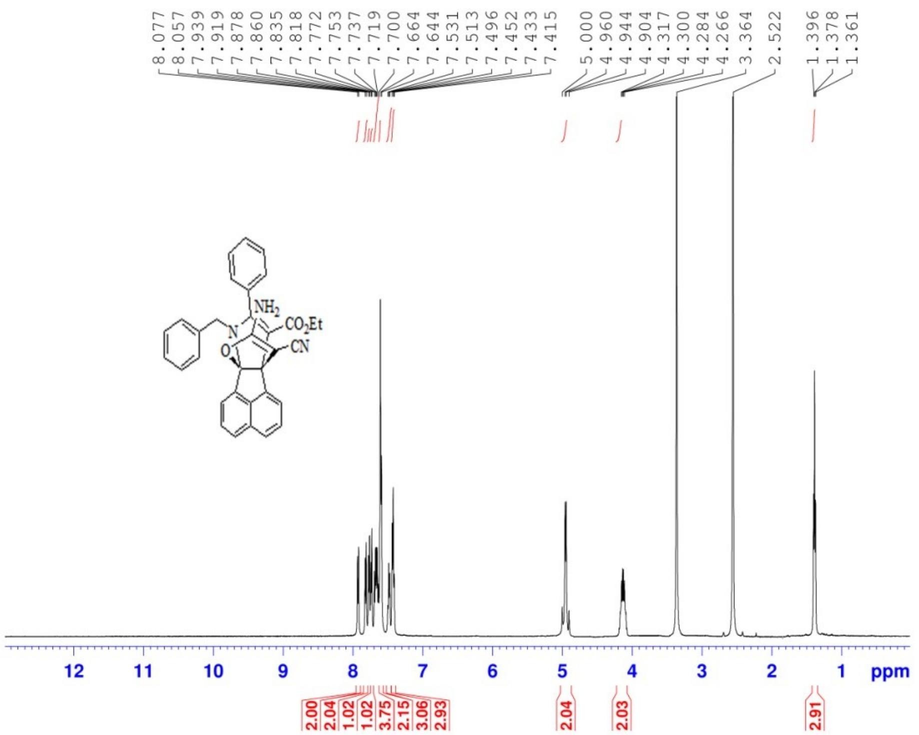

BRUKER

```
NAME       Khalij Fars UN
EXPNO                2592
PROCNO                  1
Date_            20170927
Time                12.23
INSTRUM             spect
PROBHD   5 mm PABBO BB-
PULPROG              zg30
TD                  65536
SOLVENT              DMSO
NS                     20
DS                      0
SWH             8012.820 Hz
FIDRES          0.122266 Hz
AQ             4.0894966 sec
RG                    161
DW                 62.400 usec
DE                   6.50 usec
TE                  294.9 K
D1            4.00000000 sec
TD0                     1

======== CHANNEL f1 ========
NUC1                   1H
P1                  14.00 usec
PL1                 -2.00 dB
PL1W          11.86359406 W
SFO1          400.2236020 MHz
SI                  32768
SF            400.2200000 MHz
WDW                    EM
SSB                     0
LB                   0.30 Hz
GB                      0
PC                   1.00
```

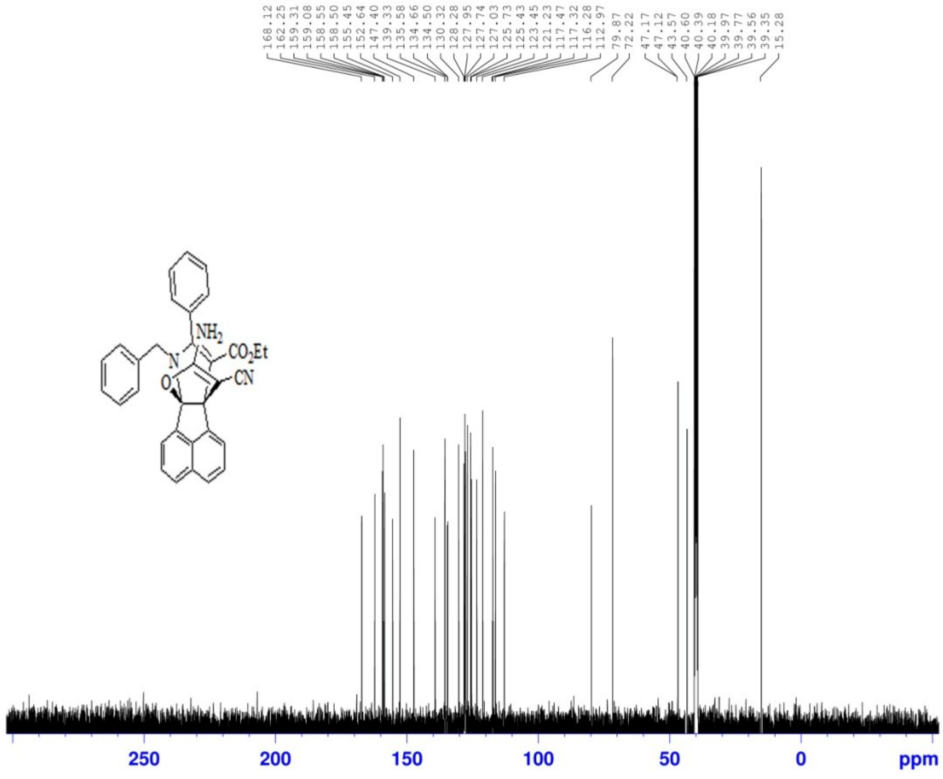

BRUKER

```
NAME       Khalij Fars UN
EXPNO                2614
PROCNO                  1
Date_            20171006
Time                16.26
INSTRUM             spect
PROBHD   5 mm PABBO BB-
PULPROG            zgpg30
TD                  65536
SOLVENT              DMSO
NS                   2020
DS                      0
SWH            35714.285 Hz
FIDRES          0.544957 Hz
AQ             0.9175540 sec
RG                   2050
DW                 14.000 usec
DE                   6.50 usec
TE                  294.5 K
D1            1.00000000 sec
D11           0.03000000 sec
TD0                     1

======== CHANNEL f1 ========
NUC1                  13C
P1                   9.00 usec
PL1                 -0.90 dB
PL1W          42.02801895 W
SFO1          100.6479784 MHz

======== CHANNEL f2 ========
CPDPRG2           waltz16
NUC2                   1H
PCPD2               90.00 usec
PL2                 -2.00 dB
PL12                14.48 dB
PL13                17.90 dB
PL2W          11.86359406 W
PL12W          0.26681873 W
PL13W          0.12139934 W
SFO2          400.2216009 MHz
SI                  32768
SF            100.6353990 MHz
WDW                    EM
SSB                     0
LB                   1.00 Hz
GB                      0
PC                   1.40
```

9-Acetyl-11-amino-7-(4-methoxybenzyl)-8-methyl-7H-6b,9a-(epoxyetheno)acenaphtho[1,2-b]pyrrole-10-carbonitrile (6d)

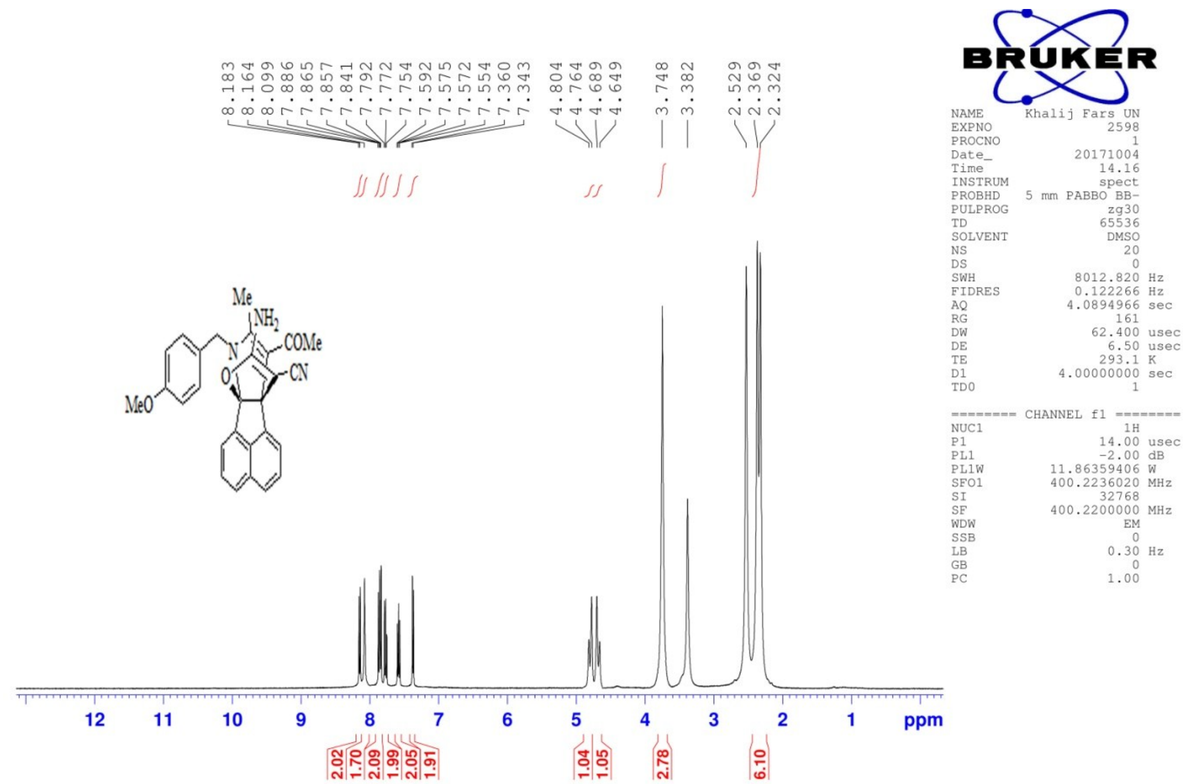

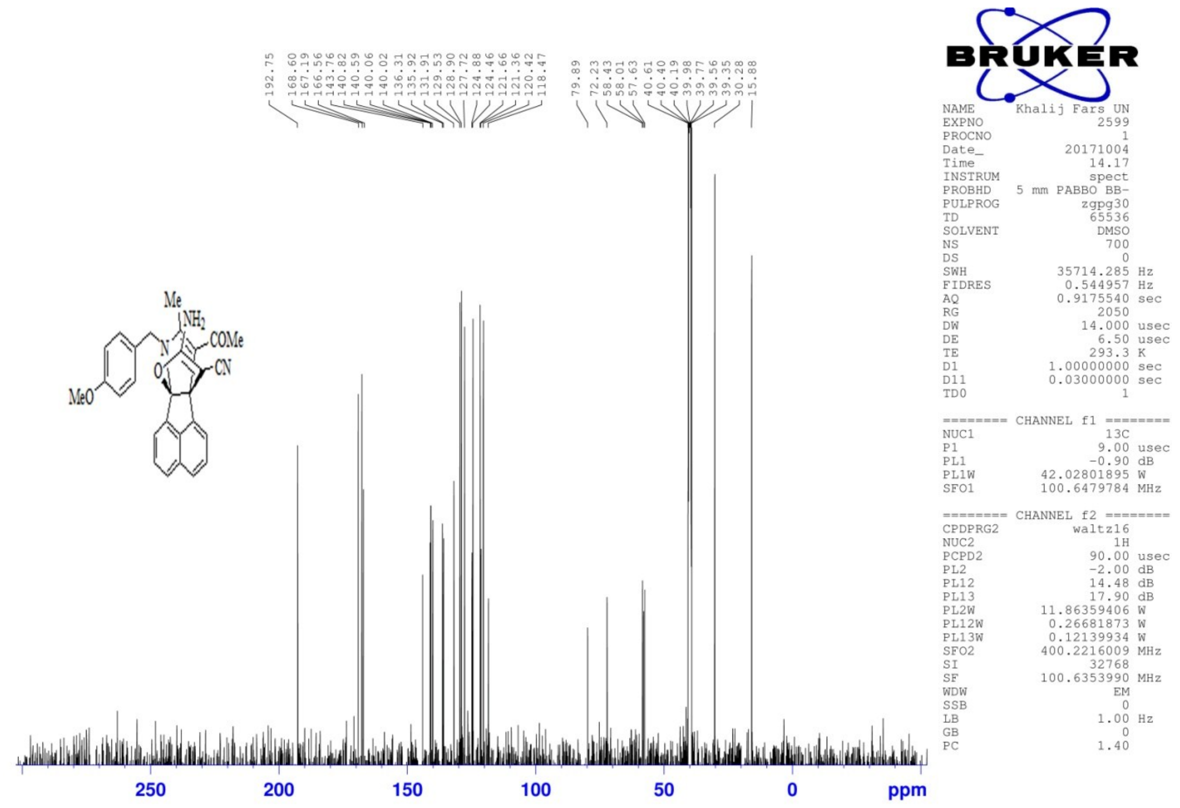

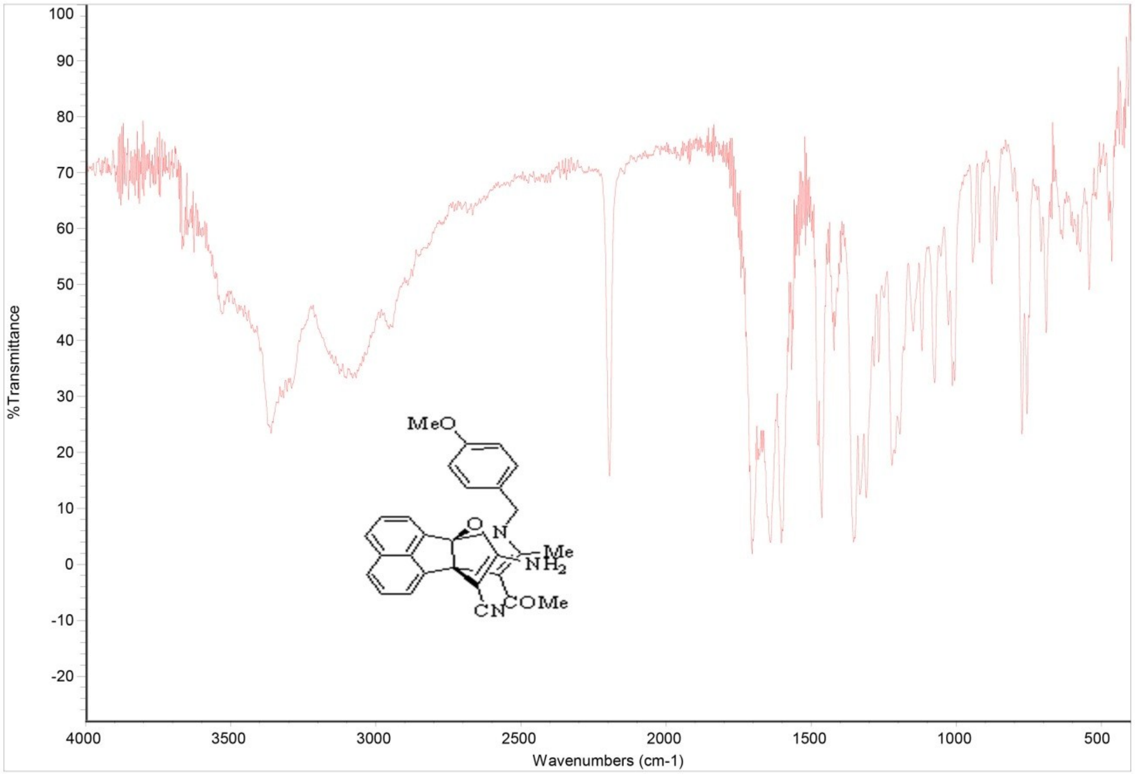

Ethyl-11-amino-10-cyano-7-(4-methoxybenzyl)-8-methyl-7H-6b,9a-(epoxyetheno)acenaphtho[1,2-b]pyrrole-9-carboxylate (6e)

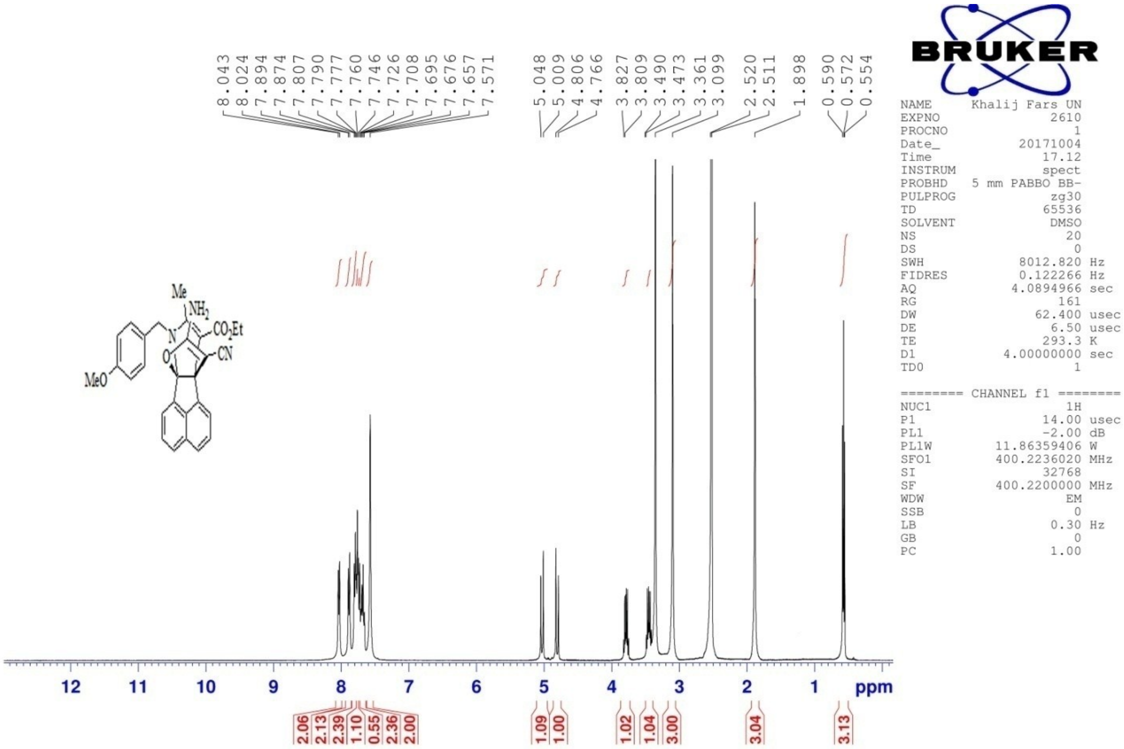

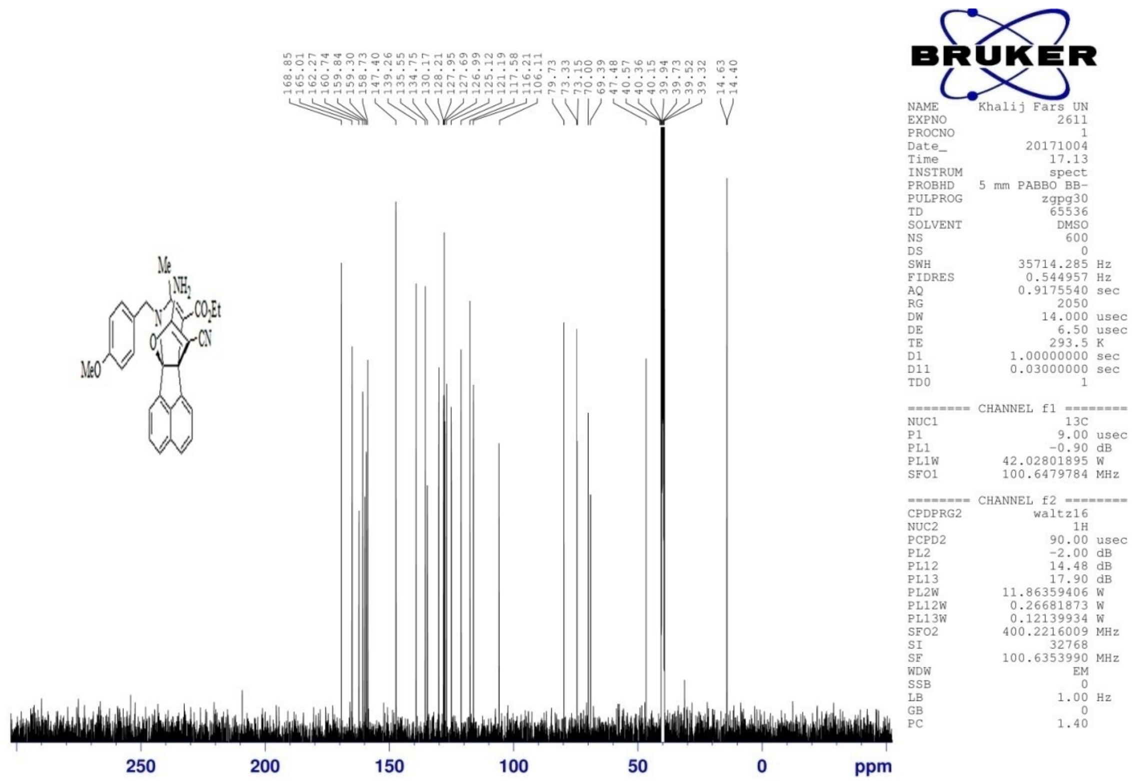

Ethyl-11-amino-7-(4-chlorobenzyl)-10-cyano-8-methyl-7H-6b,9a-(epoxyetheno)acenaphtho[1,2-b]pyrrole-9-carboxylate (6f)

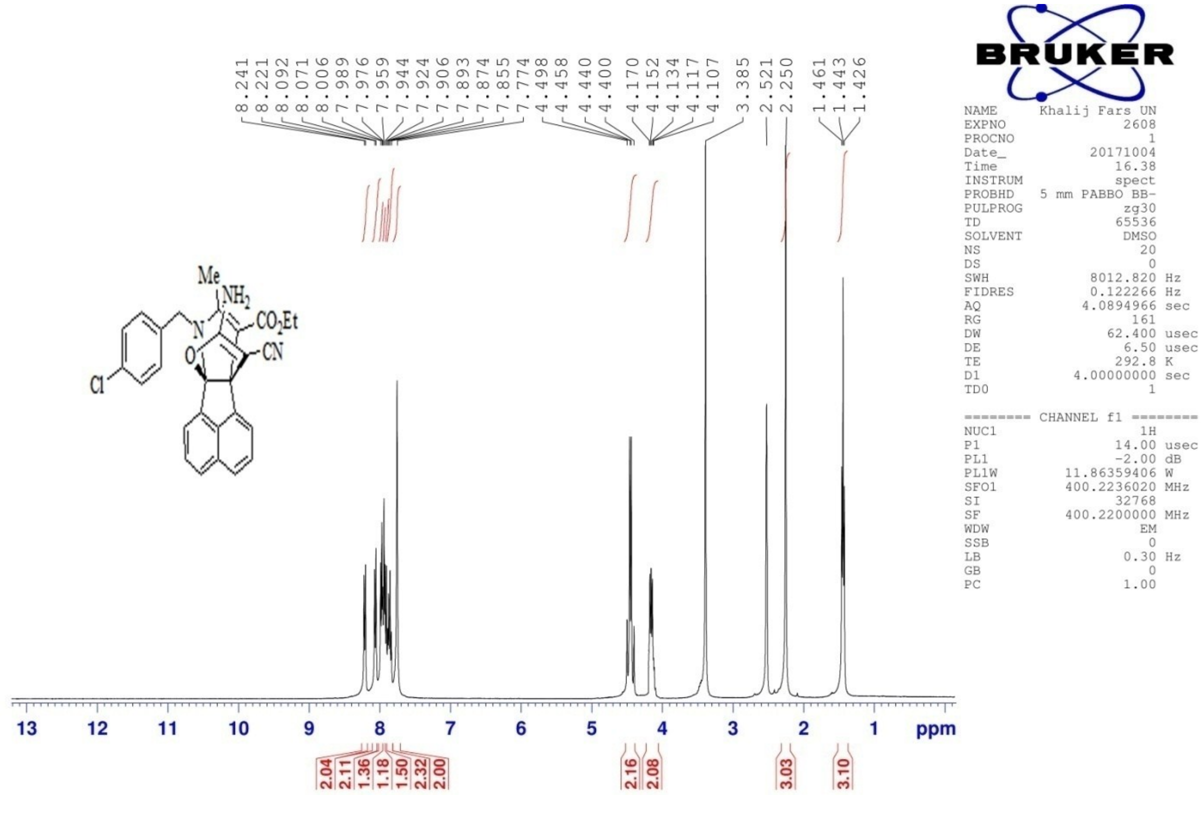

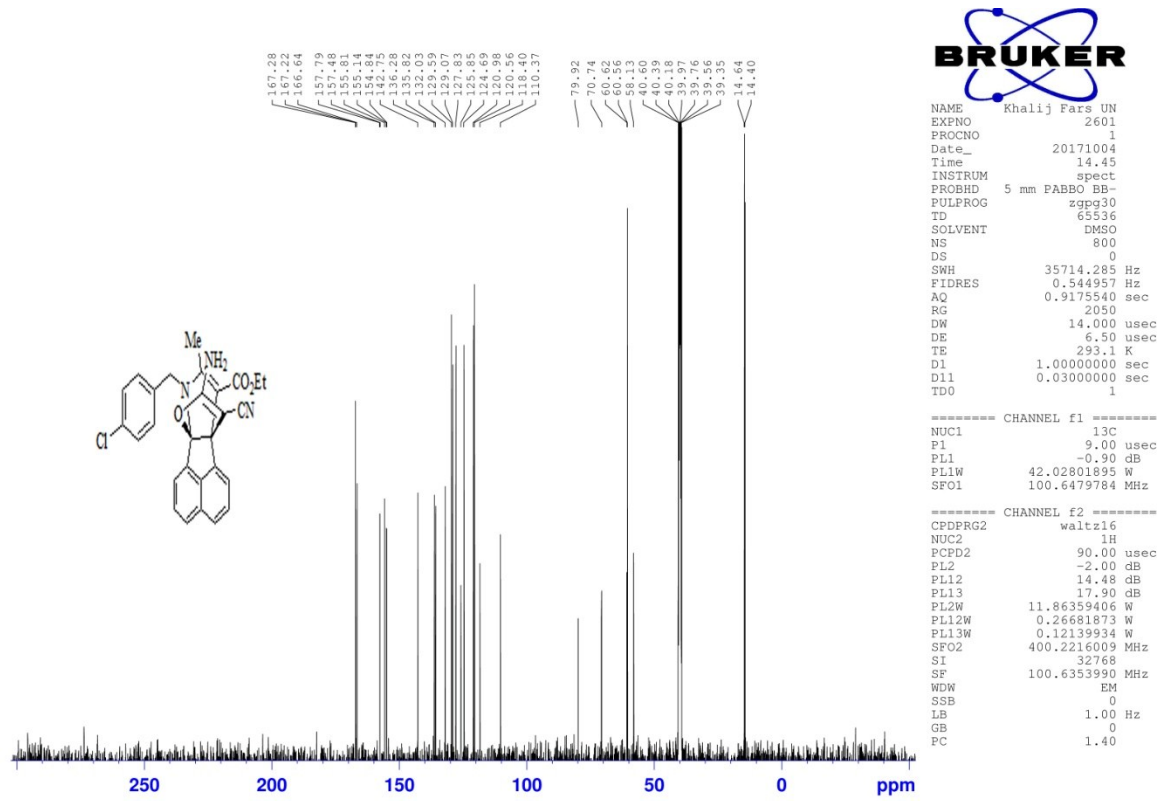

9-Acetyl-11-amino-7-(3,5-dimethylbenzyl)-8-methyl-7H-6b,9a-(epoxyetheno)acenaphtho[1,2-b]pyrrole-10-carbonitrile (6g)

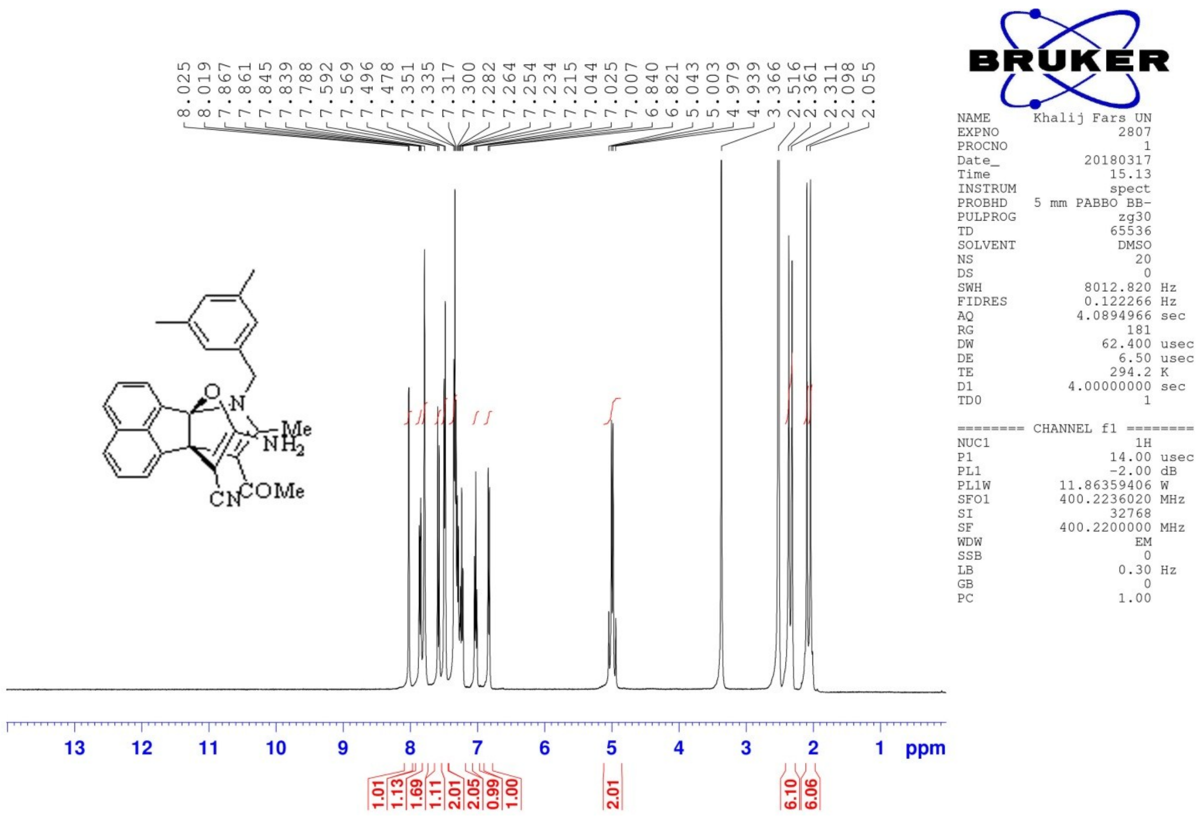

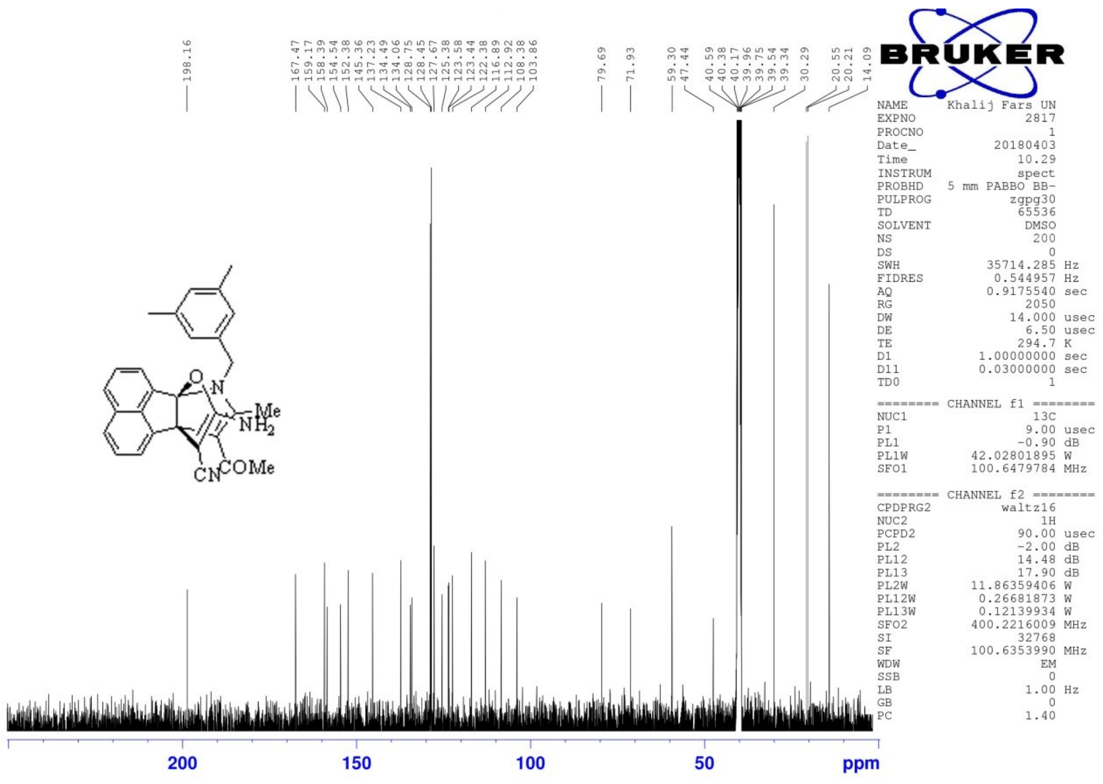

9-Acetyl-11-amino-8-methyl-7-phenyl-7H-6b,9a-(epoxyetheno)acenaphtho[1,2-b]pyrrole-10-carbonitrile (6h)

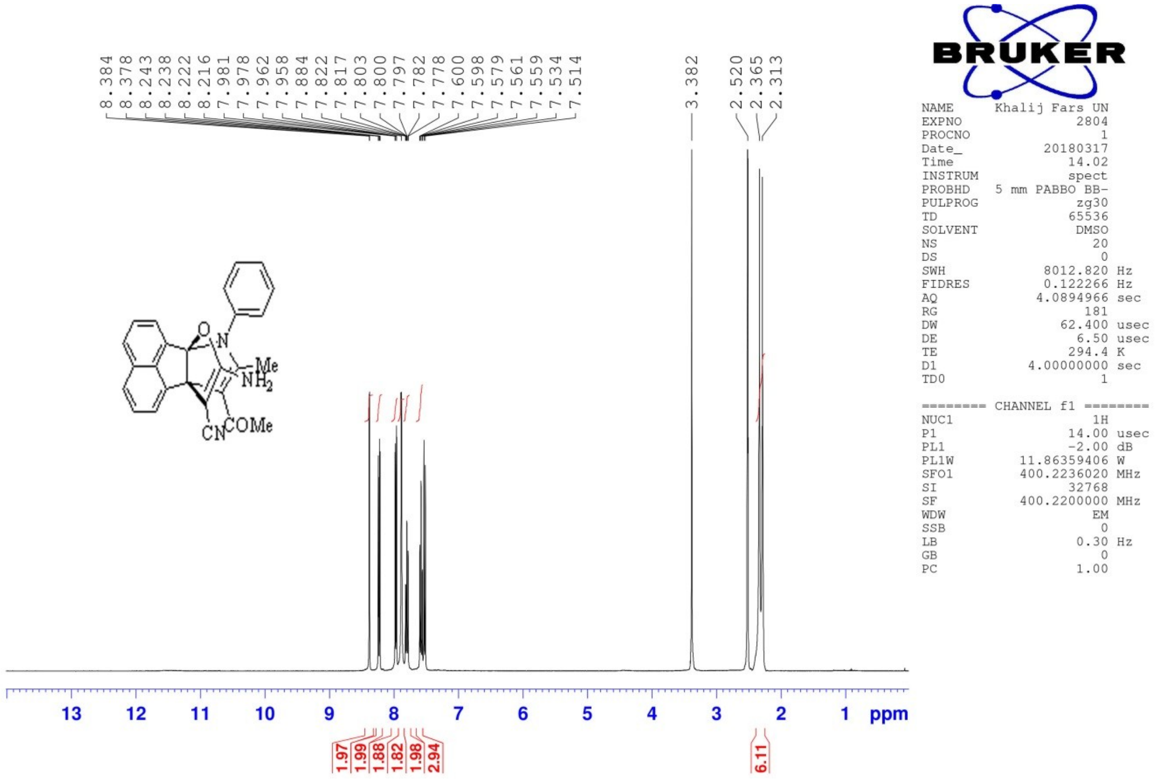

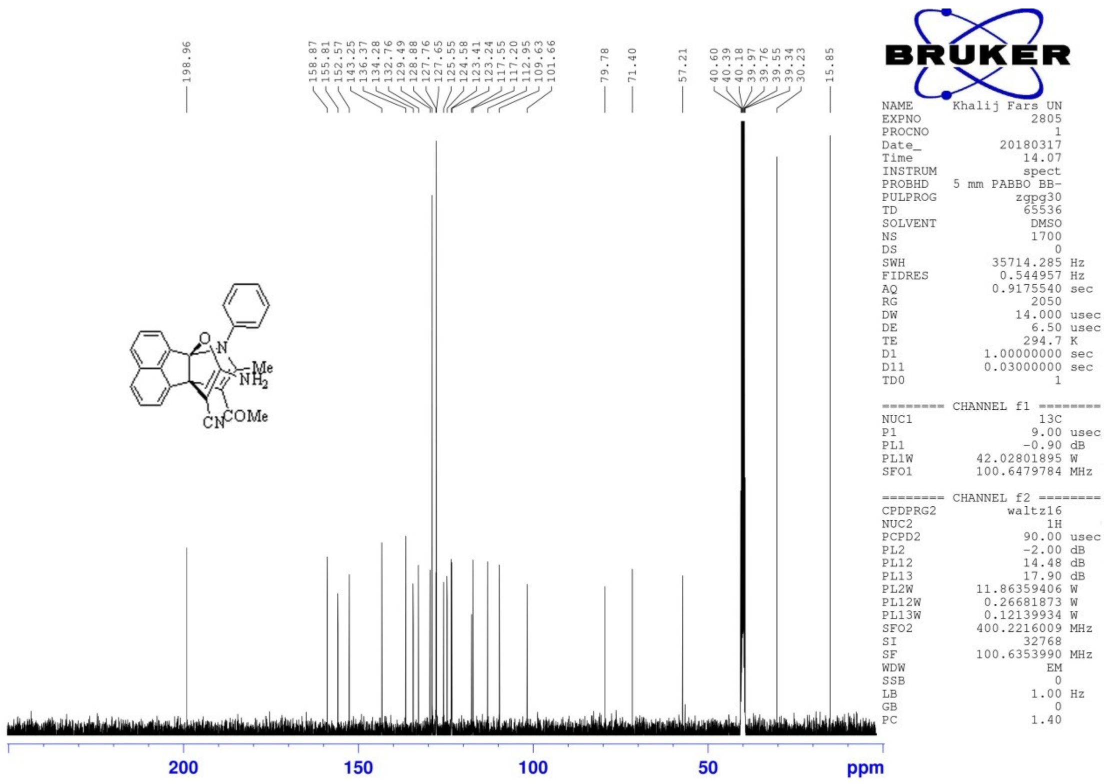

9-Acetyl-11-amino-7-(4-methoxyphenyl)-8-methyl-7H-6b,9a-(epoxyetheno)acenaphtho[1,2-b]pyrrole-10-carbonitrile (6i)

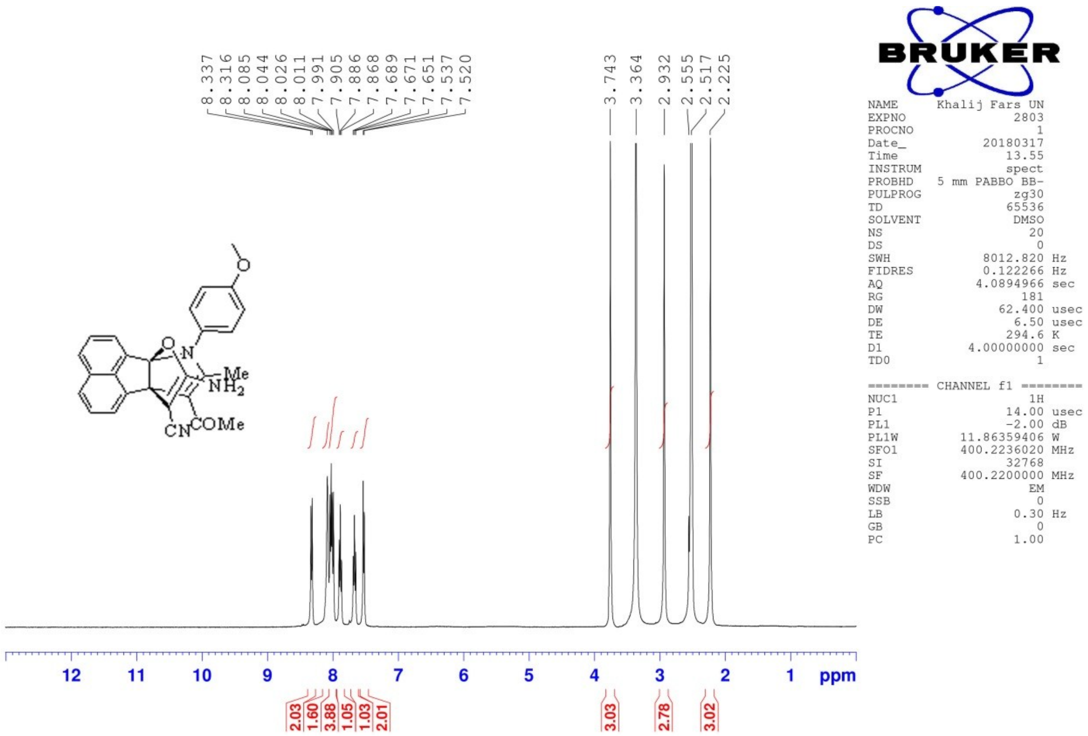

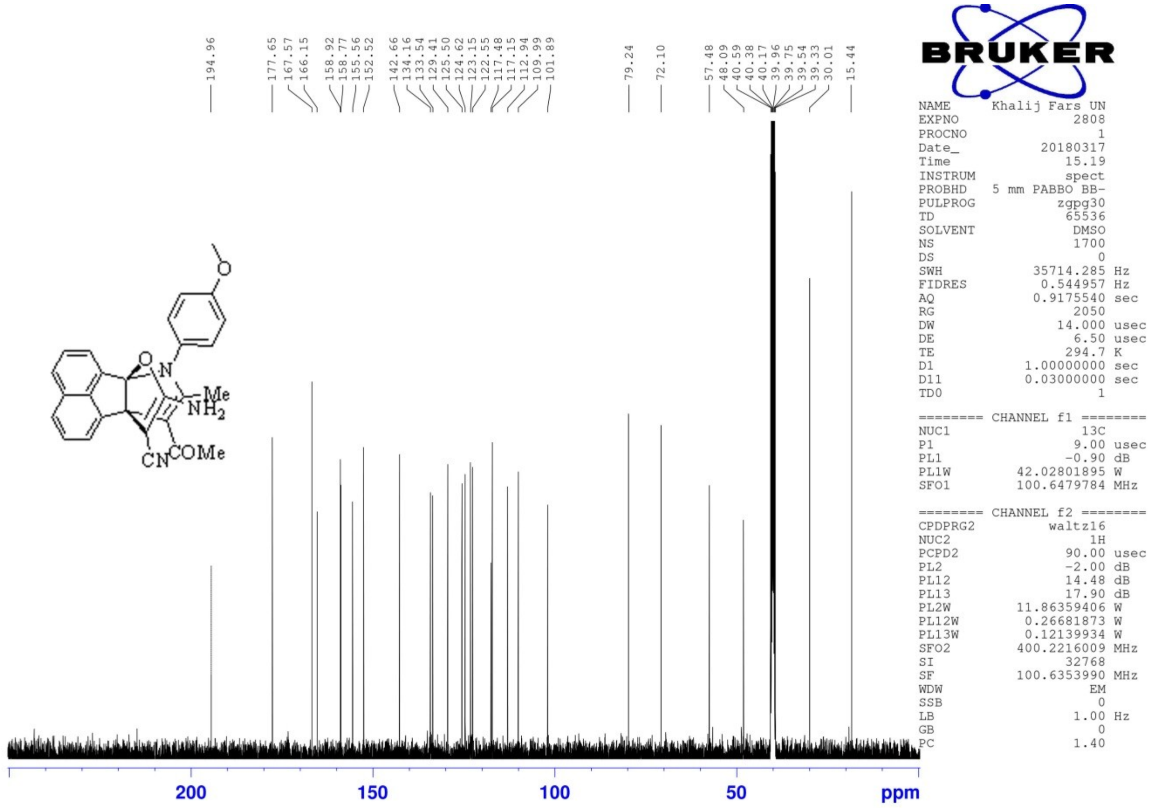

Ethyl 11-amino-10-cyano-7-(2,6-dimethylphenyl)-8-methyl-7H-6b,9a-(epoxyetheno)acenaphtho[1,2-b]pyrrole-9-carboxylate (6j)

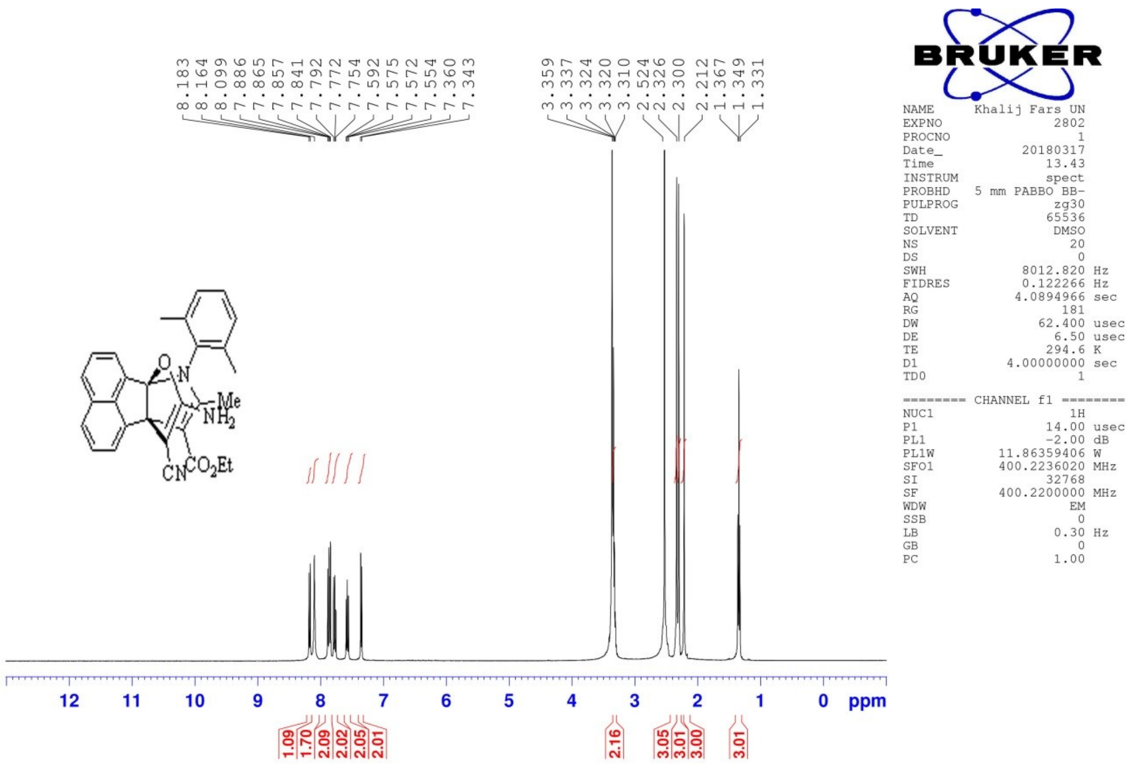

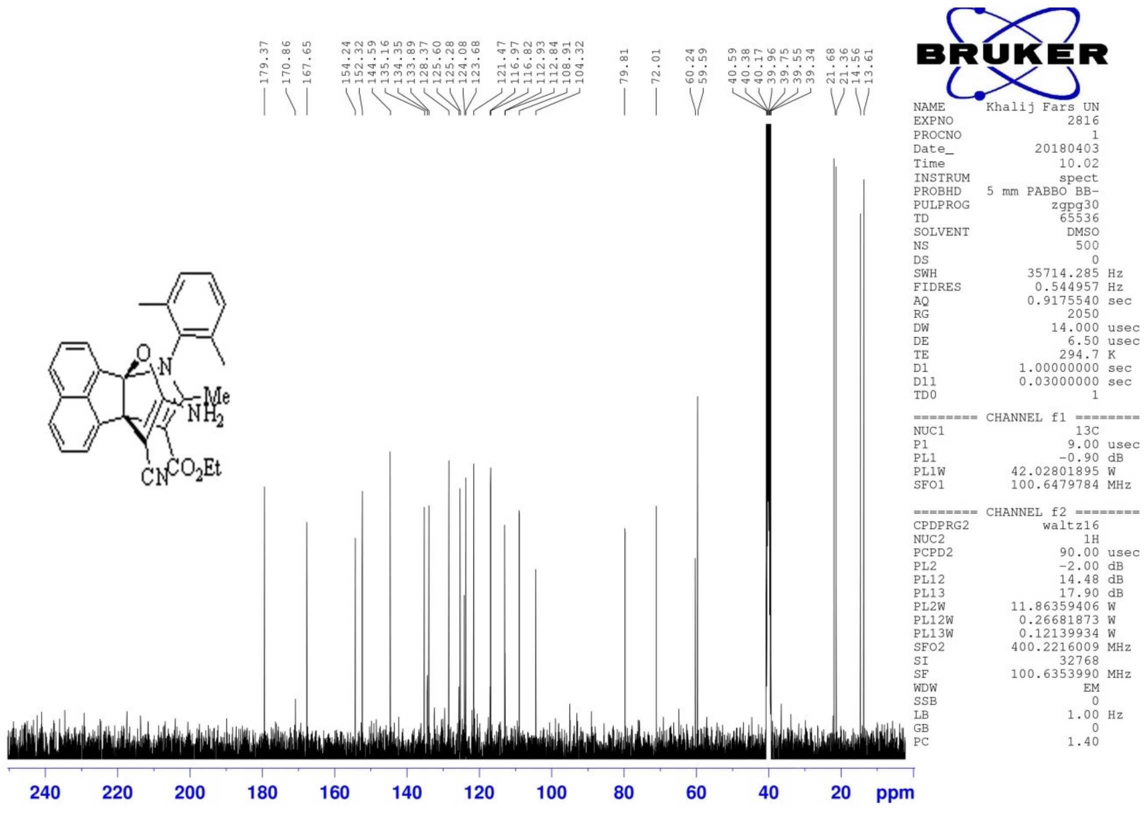

Ethyl 11-amino-7-(4-chlorophenyl)-10-cyano-8-methyl-7H-6b,9a-(epoxyetheno)acenaphtho[1,2-b]pyrrole-9-carboxylate (6k)

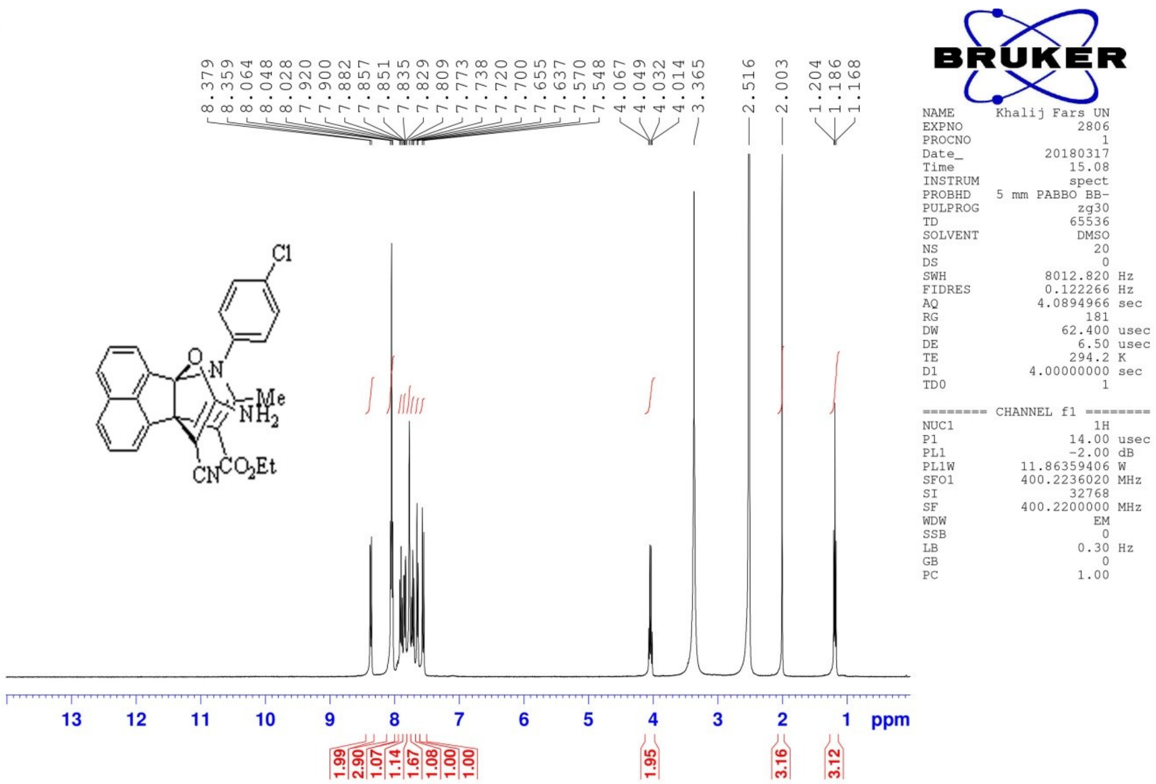
8.379 8.359 8.064 8.048 8.028 7.920 7.900 7.882 7.857 7.851 7.835 7.829 7.809 7.773 7.738 7.720 7.700 7.655 7.637 7.570 7.548 4.067 4.049 4.032 4.014 3.365 2.516 2.003 1.204 1.186 1.168
BRUKER
NAME Khalij Fars UN
EXPNO 2806
PROCNO 1
Date_ 20180317
Time 15.08
INSTRUM spect
PROBHD 5 mm PABBO BB-
PULPROG zg30
TD 65536
SOLVENT DMSO
NS 20
DS 0
SWH 8012.820 Hz
FIDRES 0.122266 Hz
AQ 4.0894966 sec
RG 181
DW 62.400 usec
DE 6.50 usec
TE 294.2 K
D1 4.00000000 sec
TD0 1
======== CHANNEL f1 ========
NUC1 1H
P1 14.00 usec
PL1 -2.00 dB
PL1W 11.86359406 W
SFO1 400.2236020 MHz
SI 32768
SF 400.2200000 MHz
WDW EM
SSB 0
LB 0.30 Hz
GB 0
PC 1.00
Cl
O
N
Me
NH2
CN
CO2Et
13 12 11 10 9 8 7 6 5 4 3 2 1 ppm
1.99 2.90 1.07 1.14 1.67 1.08 1.00 1.00 1.95 3.16 3.12

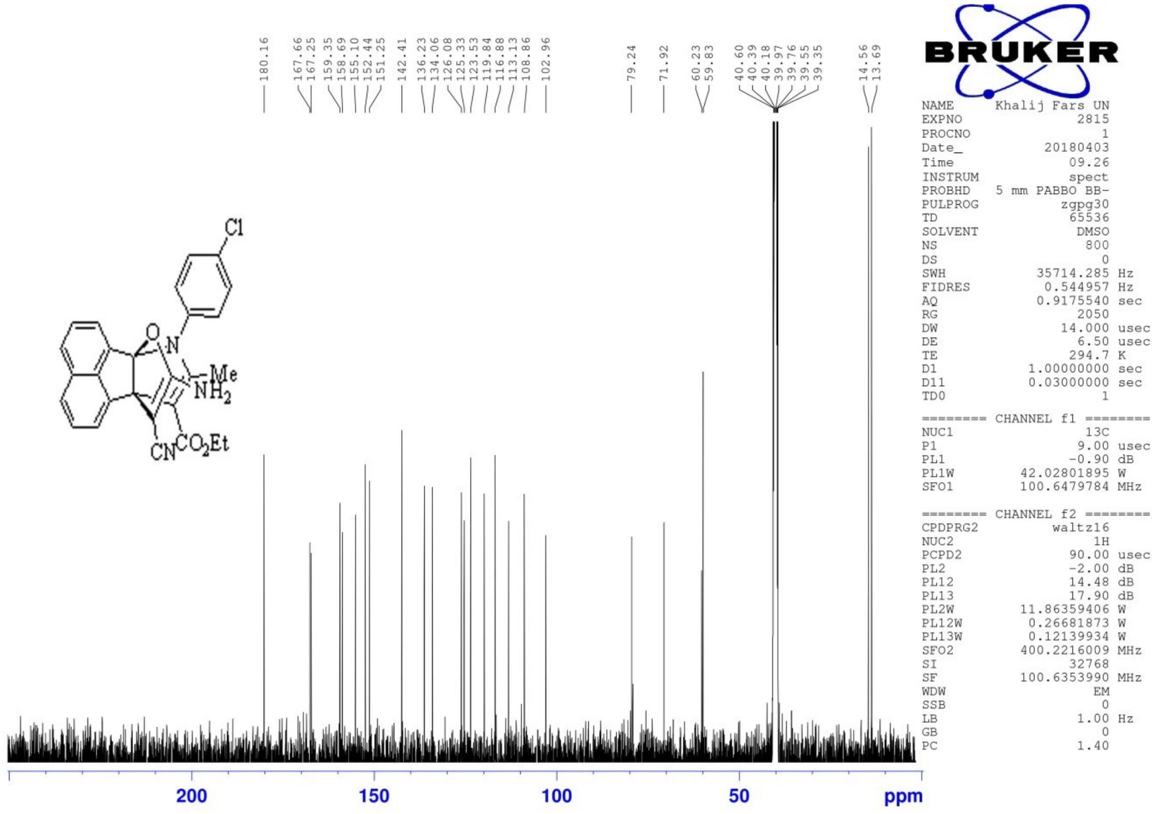
180.16 167.66 167.25 159.35 158.69 155.10 152.44 151.25 142.41 136.23 134.06 126.08 125.33 123.53 119.84 116.88 113.13 108.86 102.96 79.24 71.92 60.23 59.83 40.60 40.39 40.18 39.97 39.76 39.55 39.35 14.56 13.69
BRUKER
NAME Khalij Fars UN
EXPNO 2815
PROCNO 1
Date_ 20180403
Time 09.26
INSTRUM spect
PROBHD 5 mm PABBO BB-
PULPROG zgpg30
TD 65536
SOLVENT DMSO
NS 800
DS 0
SWH 35714.285 Hz
FIDRES 0.544957 Hz
AQ 0.9175540 sec
RG 2050
DW 14.000 usec
DE 6.50 usec
TE 294.7 K
D1 1.00000000 sec
D11 0.03000000 sec
TD0 1
======== CHANNEL f1 ========
NUC1 13C
P1 9.00 usec
PL1 -0.90 dB
PL1W 42.02801895 W
SFO1 100.6479784 MHz
======== CHANNEL f2 ========
CPDPRG2 waltz16
NUC2 1H
PCPD2 90.00 usec
PL2 -2.00 dB
PL12 14.48 dB
PL13 17.90 dB
PL2W 11.86359406 W
PL12W 0.26681873 W
PL13W 0.12139934 W
SFO2 400.2216009 MHz
SI 32768
SF 100.6353990 MHz
WDW EM
SSB 0
LB 1.00 Hz
GB 0
PC 1.40
Cl
O
N
Me
NH2
CN
CO2Et
200 150 100 50 ppm

Ethyl-11-amino-10-cyano-7-ethyl-8-methyl-7H-6b,9a-(epoxyetheno)acenaphtho[1,2-b]pyrrole-9-carboxylate (6l)

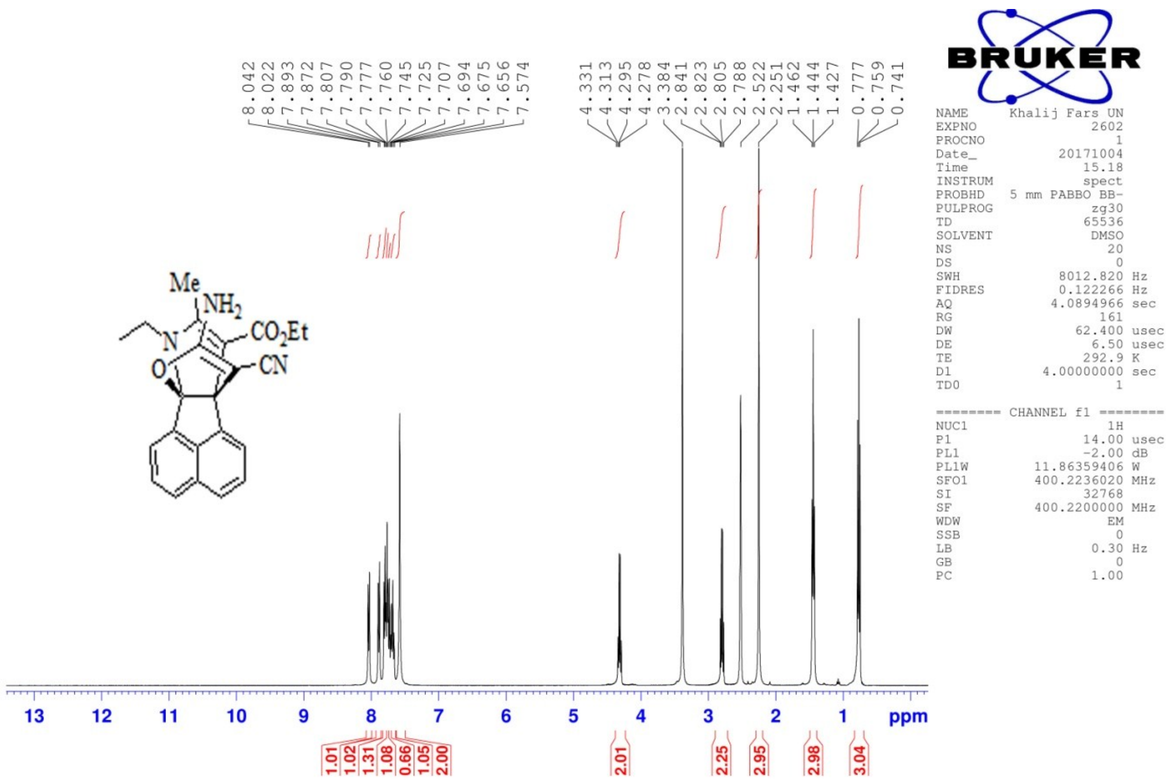

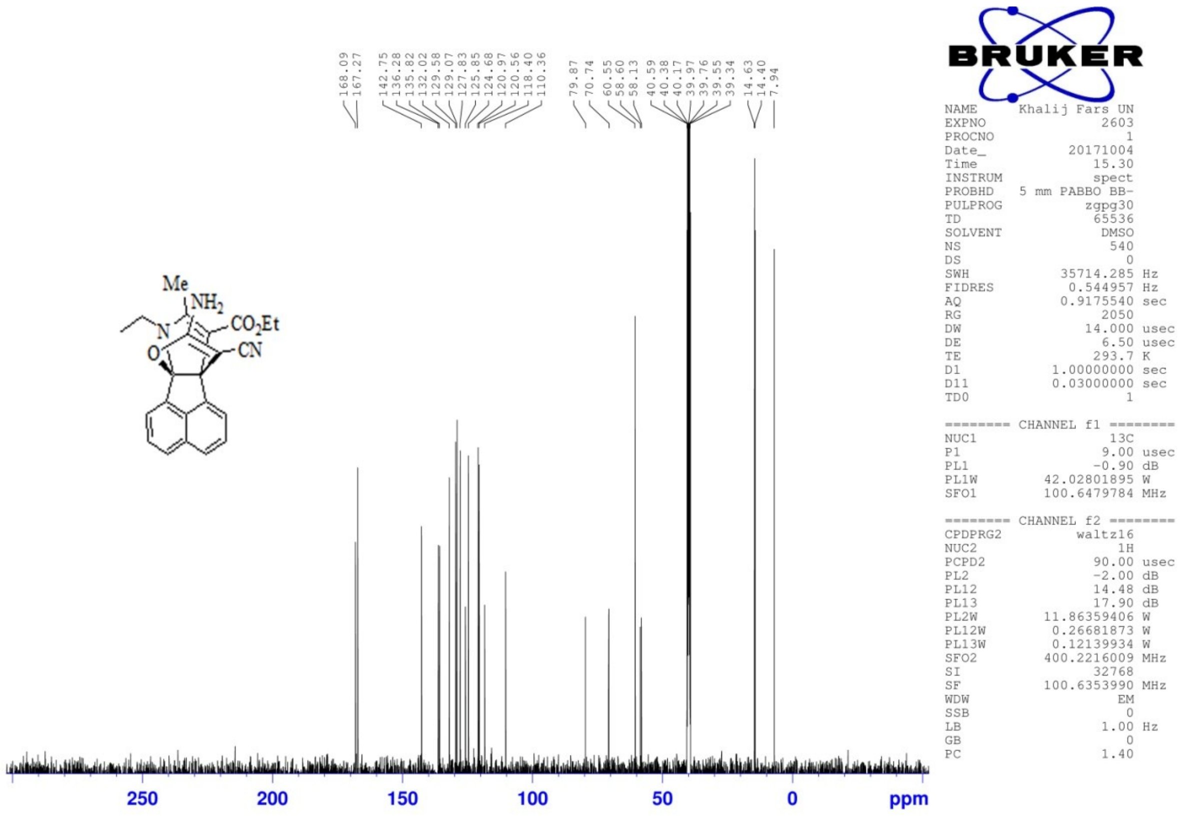

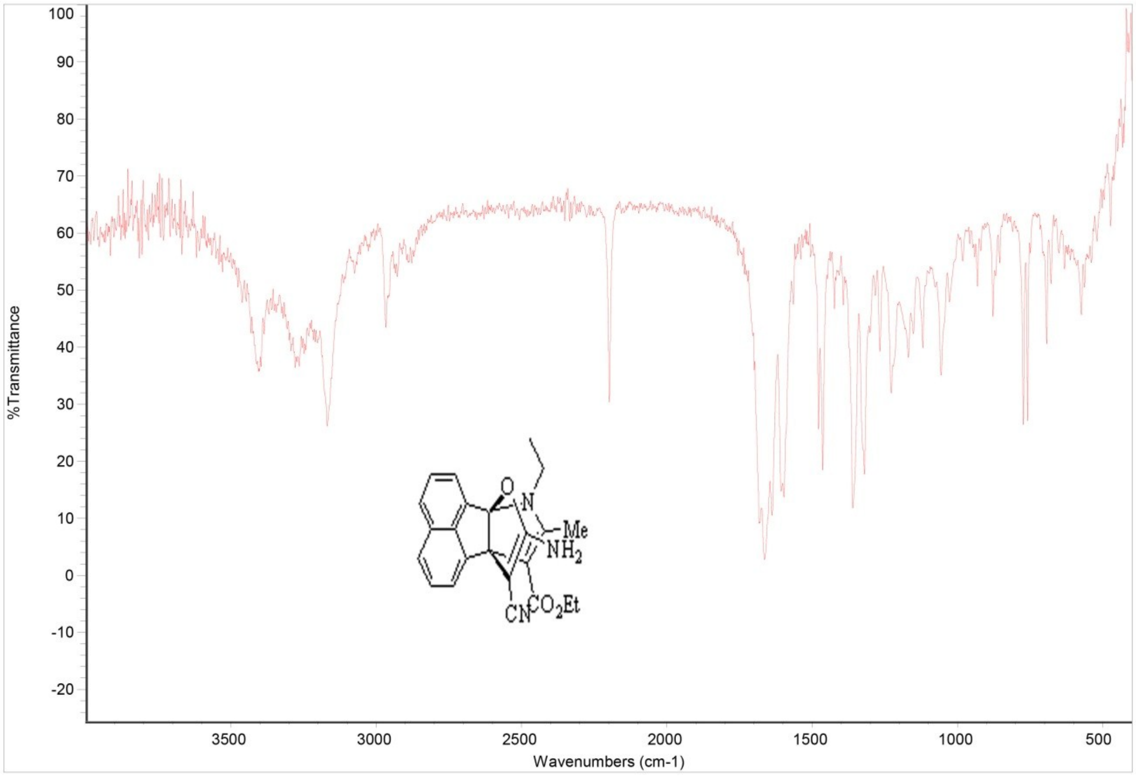

9-Acetyl-11-amino-7-ethyl-8-methyl-7H-6b,9a-(epoxyetheno)acenaphtho[1,2-b]pyrrole-10-carbonitrile (6m)

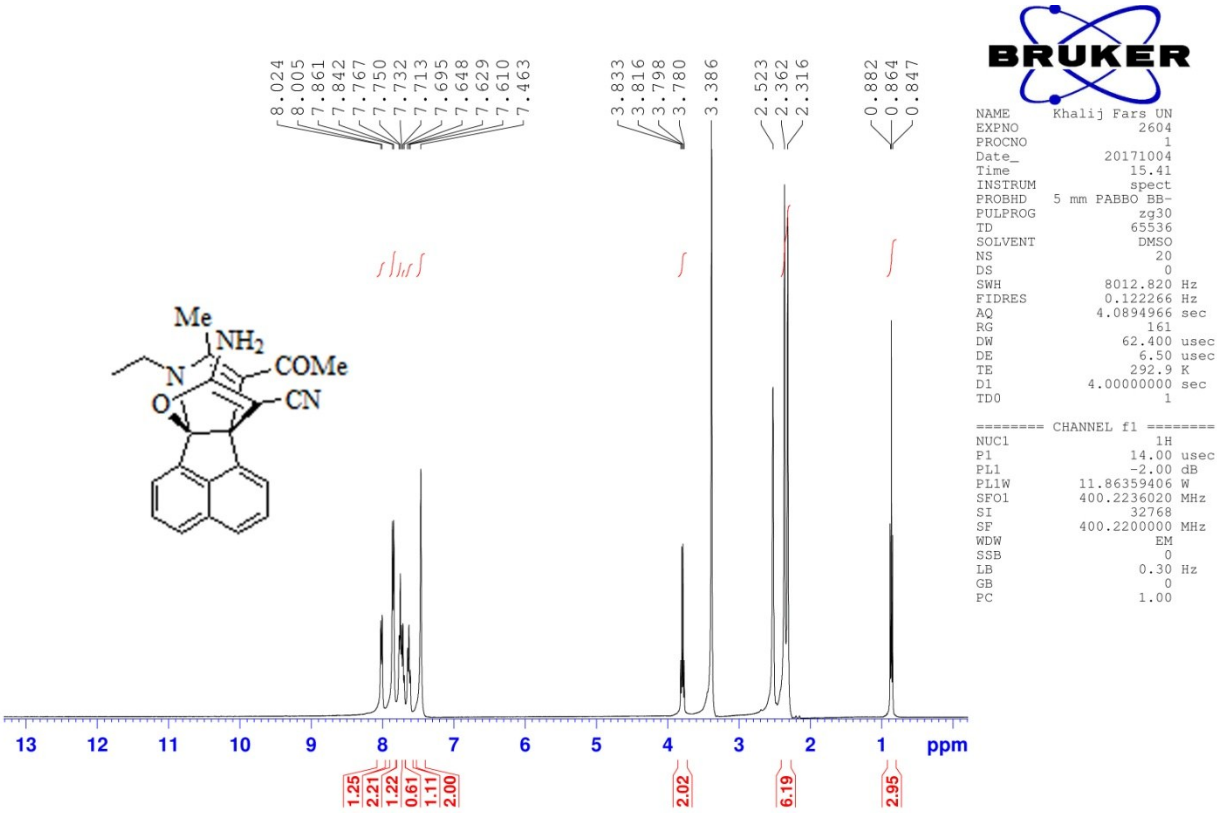

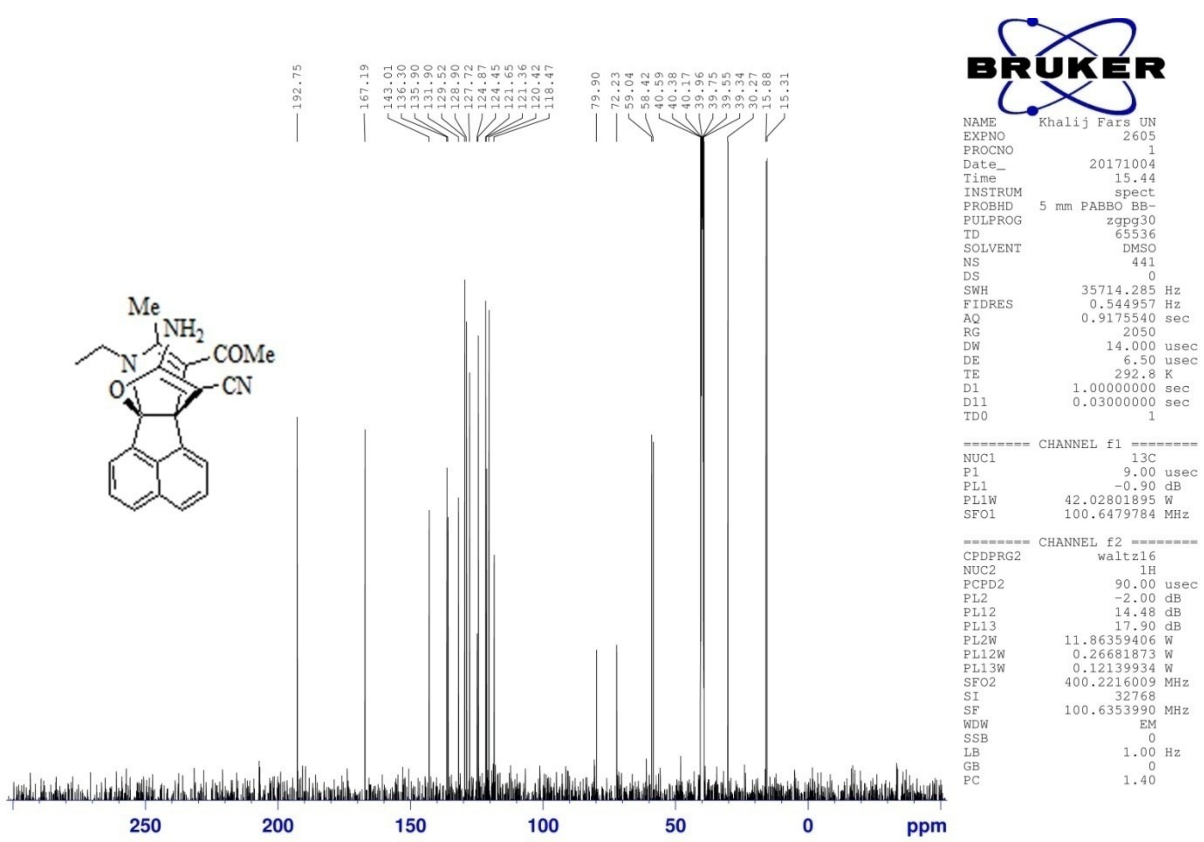

Ethyl-11-amino-7-butyl-10-cyano-8-methyl-7H-6b,9a-(epoxyetheno)acenaphtho[1,2-b]pyrrole-9-carboxylate (6n)

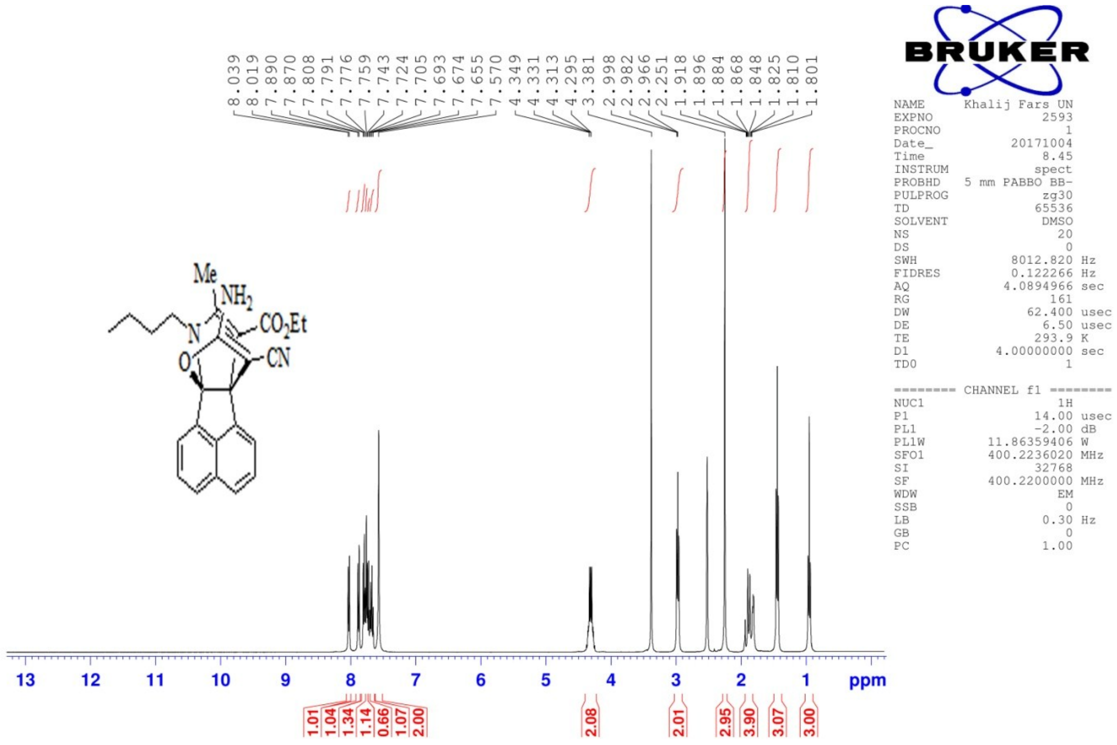

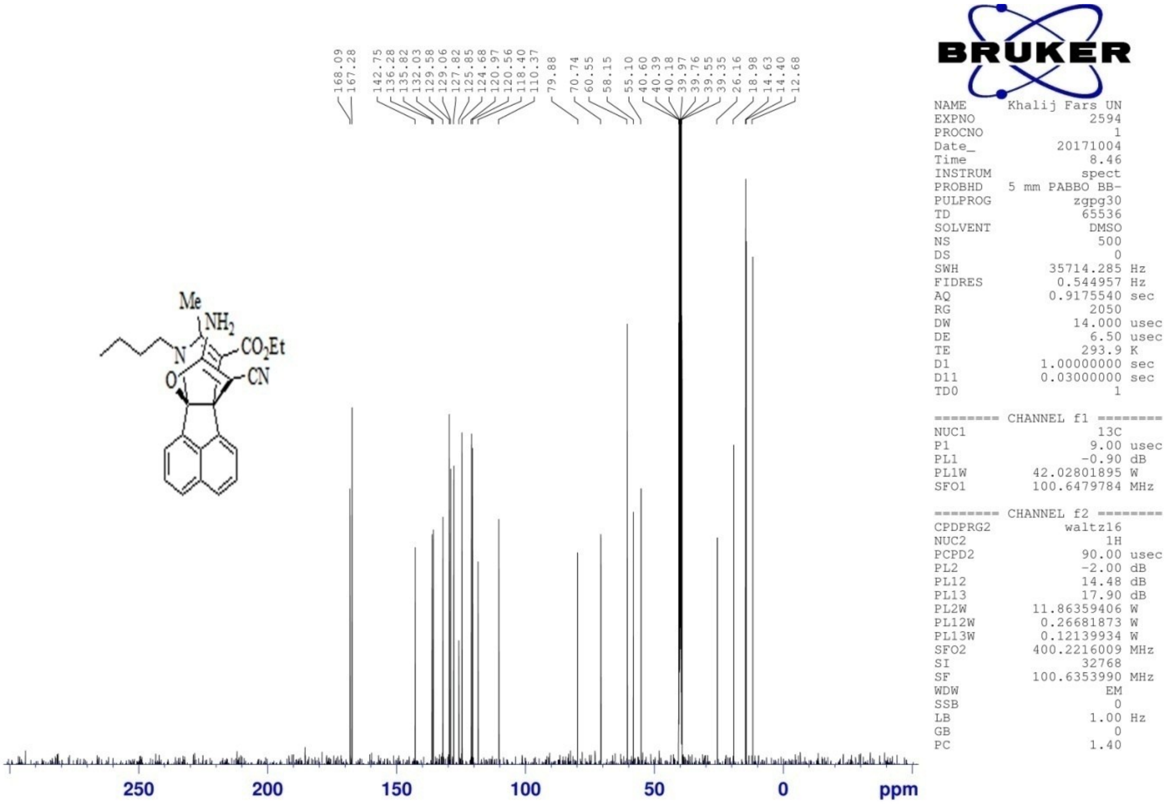
168.09
167.28
142.75
136.28
135.82
132.03
129.58
129.06
127.82
125.85
124.68
120.97
120.56
118.40
110.37
79.88
70.74
60.55
58.15
55.10
40.60
40.39
40.18
39.97
39.76
39.55
39.35
26.16
18.98
14.63
14.40
12.68
Me
NH_2
N
CO_2Et
O
CN
BRUKER
NAME Khalij Fars UN
EXPNO 2594
PROCNO 1
Date_ 20171004
Time 8.46
INSTRUM spect
PROBHD 5 mm PABBO BB-
PULPROG zgpg30
TD 65536
SOLVENT DMSO
NS 500
DS 0
SWH 35714.285 Hz
FIDRES 0.544957 Hz
AQ 0.9175540 sec
RG 2050
DW 14.000 usec
DE 6.50 usec
TE 293.9 K
D1 1.00000000 sec
D11 0.03000000 sec
TD0 1
======== CHANNEL f1 ========
NUC1 13C
P1 9.00 usec
PL1 -0.90 dB
PL1W 42.02801895 W
SFO1 100.6479784 MHz
======== CHANNEL f2 ========
CPDPRG2 waltz16
NUC2 1H
PCPD2 90.00 usec
PL2 -2.00 dB
PL12 14.48 dB
PL13 17.90 dB
PL2W 11.86359406 W
PL12W 0.26681873 W
PL13W 0.12139934 W
SFO2 400.2216009 MHz
SI 32768
SF 100.6353990 MHz
WDW EM
SSB 0
LB 1.00 Hz
GB 0
PC 1.40
250
200
150
100
50
0
ppm

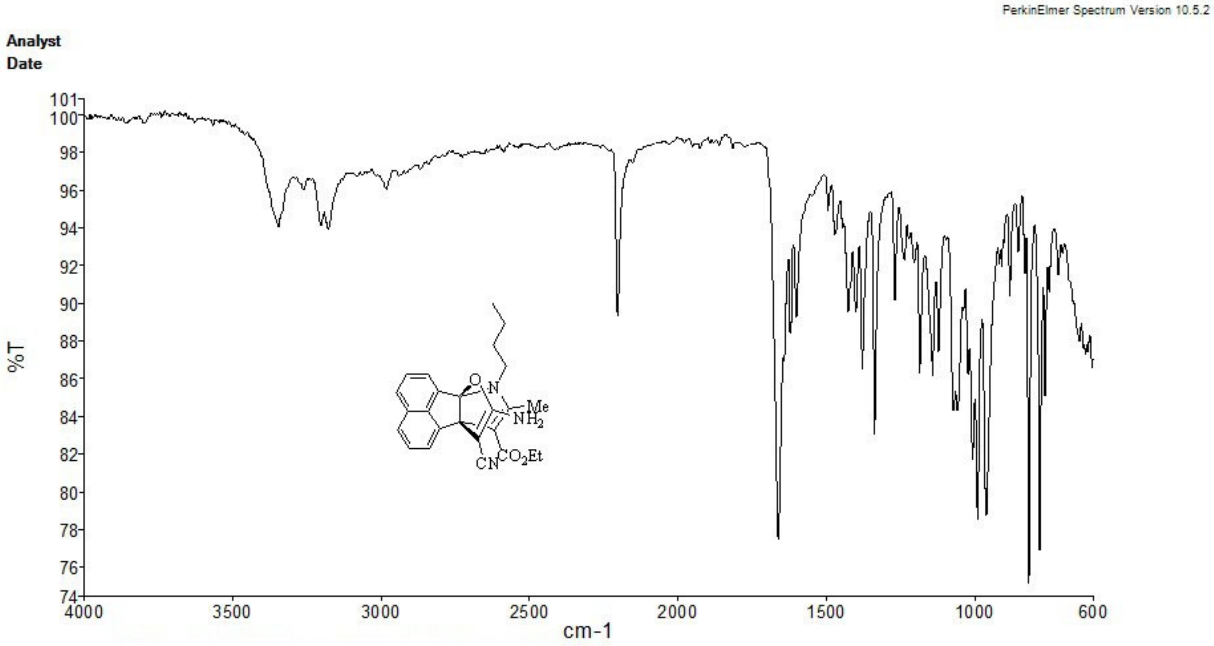
PerkinElmer Spectrum Version 10.5.2
Analyst
Date
101
100
98
96
94
92
90
88
86
84
82
80
78
76
74
%T
O
N
Me
NH_2
CN
CO_2Et
4000
3500
3000
2500
2000
1500
1000
600
cm-1

9-Acetyl-11-amino-7-butyl-8-methyl-7H-6b,9a-(epoxyetheno)acenaphtho[1,2-b]pyrrole-10-carbonitrile (6o)

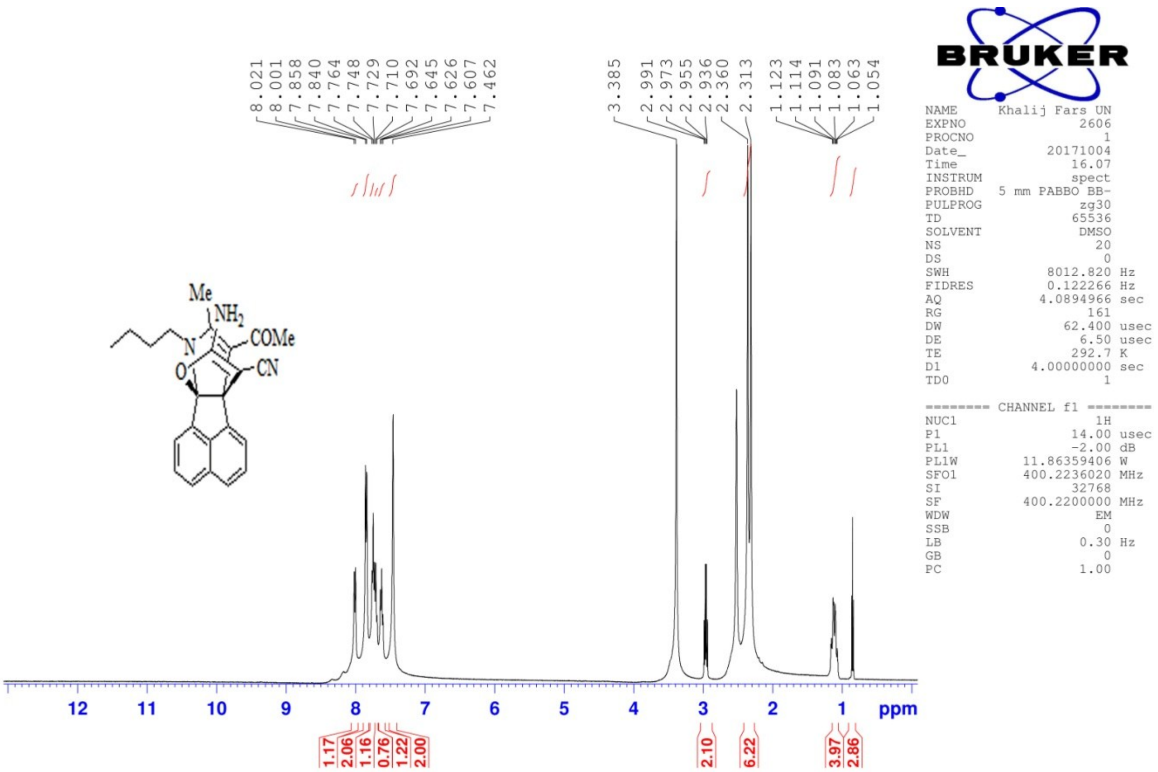

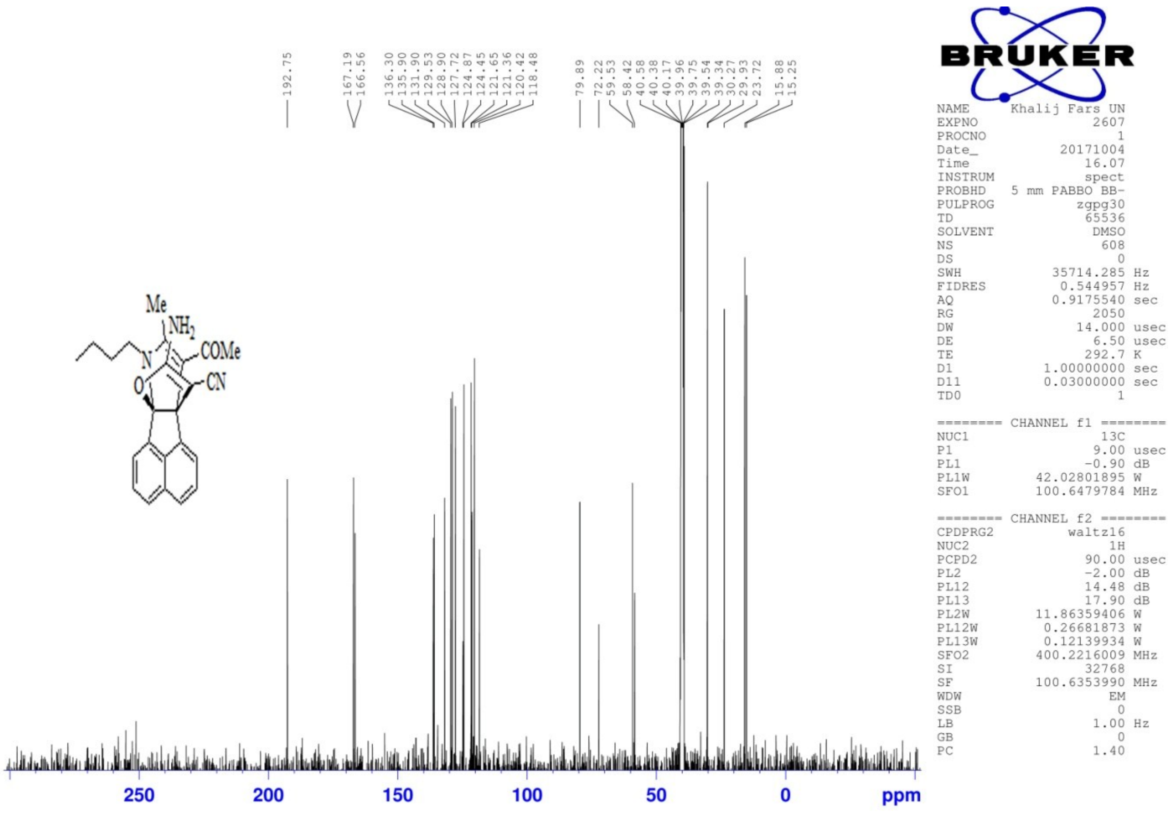

Ethyl-9-acetyl-11-amino-7-benzyl-8-methyl-7H-6b,9a-(epoxyetheno)acenaphtho[1,2-b]pyrrole-10-carboxylate (6p)

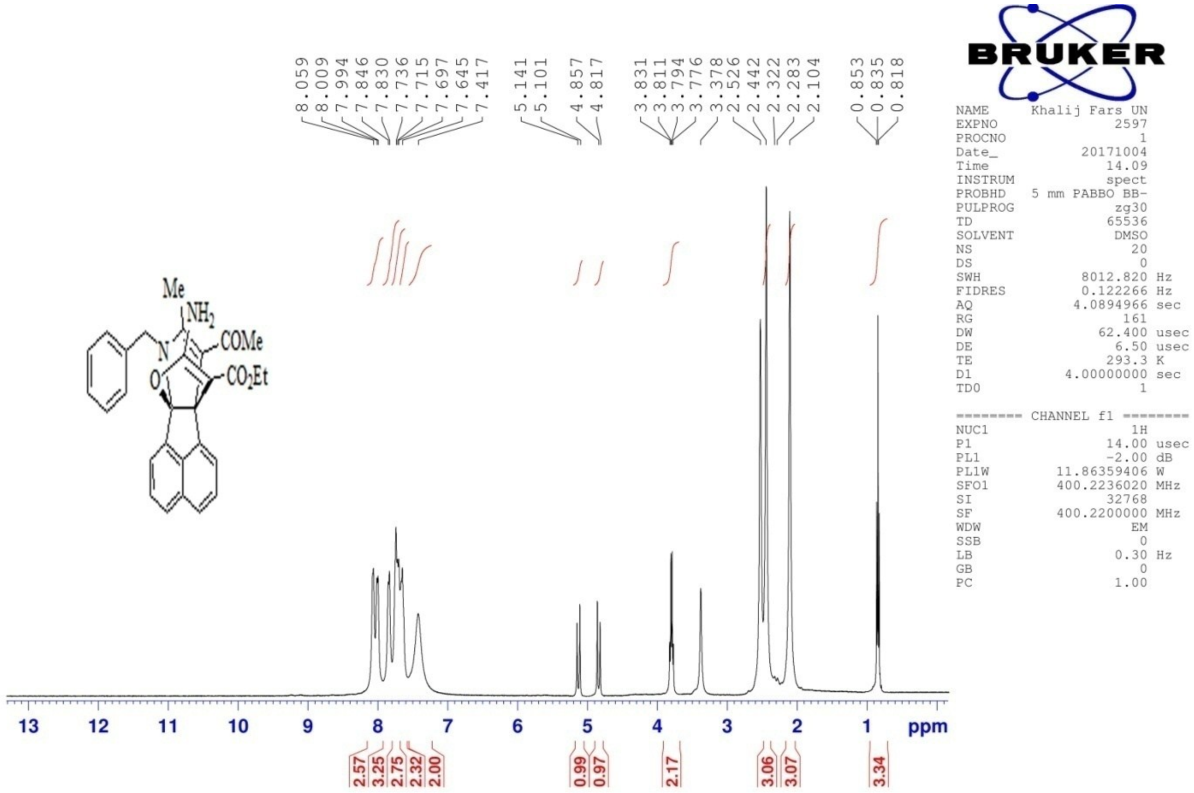

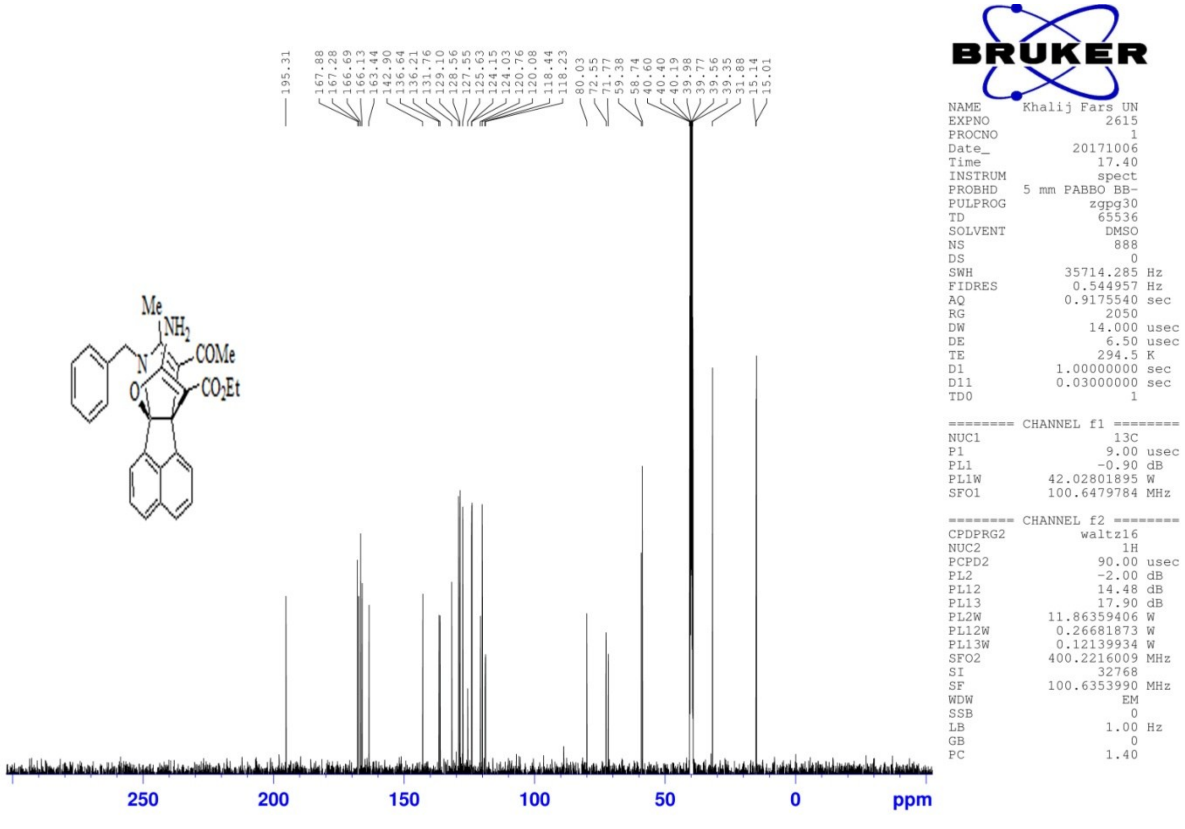

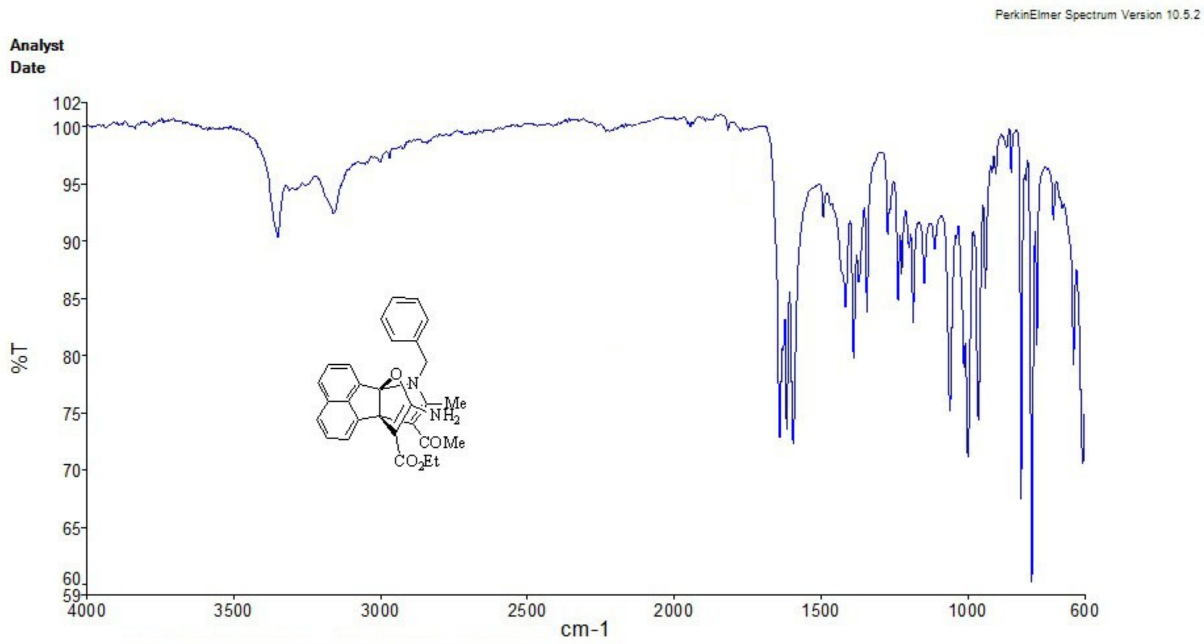

9-Ethyl-10-methyl-11-amino-7-ethyl-8-methyl-7H-6b,9a-(epoxyetheno)acenaphtho[1,2-b]pyrrole-9,10-dicarboxylate (6q)

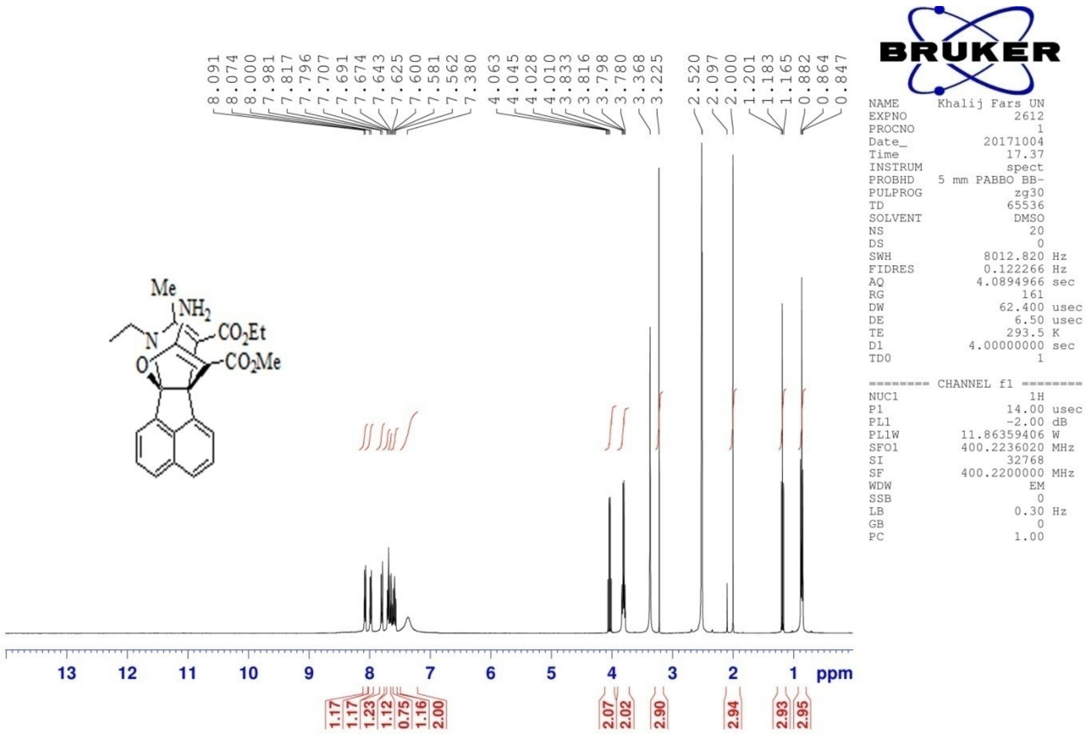

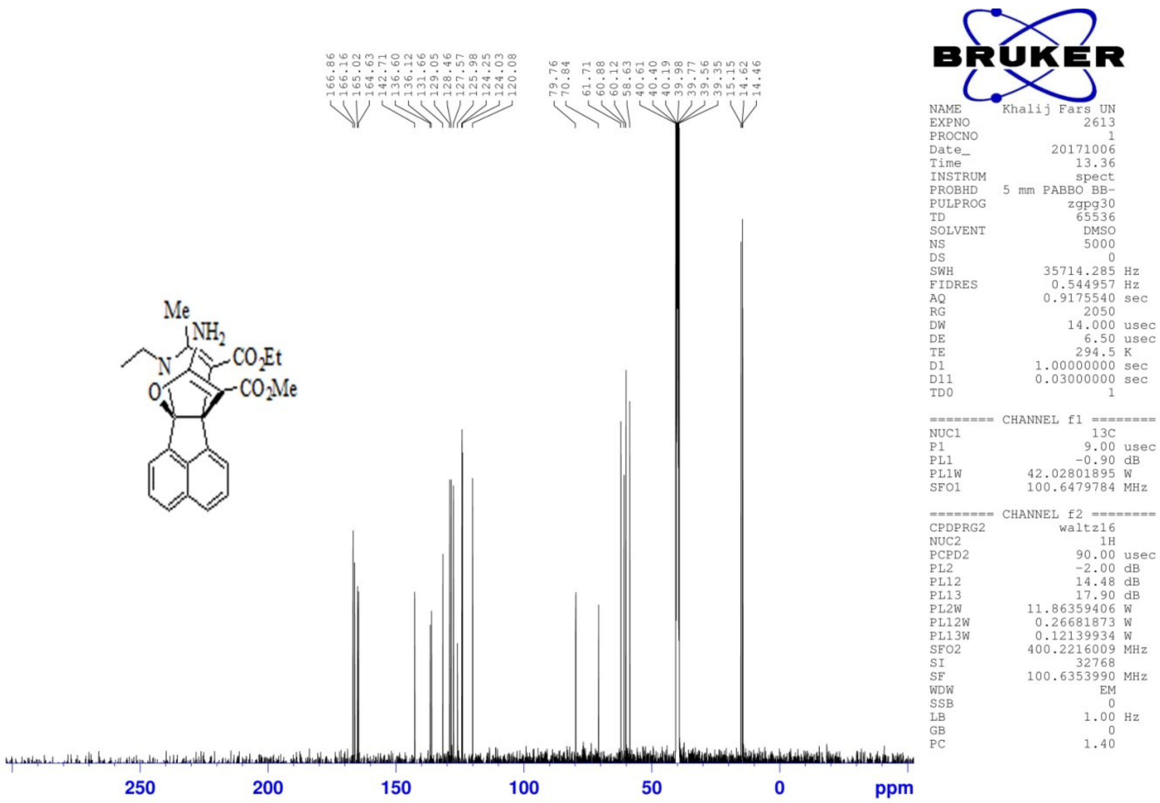

166.86
166.16
165.02
164.63
142.71
136.60
136.12
131.66
129.05
128.46
127.57
125.98
124.25
124.03
120.08
79.76
70.84
61.71
60.88
60.12
58.63
40.61
40.40
40.19
39.98
39.77
39.56
39.35
15.15
14.62
14.46
Me
NH_2
N
CO_2Et
O
CO_2Me
250
200
150
100
50
0
ppm
BRUKER
NAME Khalij Fars UN
EXPNO 2613
PROCNO 1
Date_ 20171006
Time 13.36
INSTRUM spect
PROBHD 5 mm PABBO BB-
PULPROG zgpg30
TD 65536
SOLVENT DMSO
NS 5000
DS 0
SWH 35714.285 Hz
FIDRES 0.544957 Hz
AQ 0.9175540 sec
RG 2050
DW 14.000 usec
DE 6.50 usec
TE 294.5 K
D1 1.00000000 sec
D11 0.03000000 sec
TD0 1
======== CHANNEL f1 ========
NUC1 13C
P1 9.00 usec
PL1 -0.90 dB
PL1W 42.02801895 W
SFO1 100.6479784 MHz
======== CHANNEL f2 ========
CPDPRG2 waltz16
NUC2 1H
PCPD2 90.00 usec
PL2 -2.00 dB
PL12 14.48 dB
PL13 17.90 dB
PL2W 11.86359406 W
PL12W 0.26681873 W
PL13W 0.12139934 W
SFO2 400.2216009 MHz
SI 32768
SF 100.6353990 MHz
WDW EM
SSB 0
LB 1.00 Hz
GB 0
PC 1.40

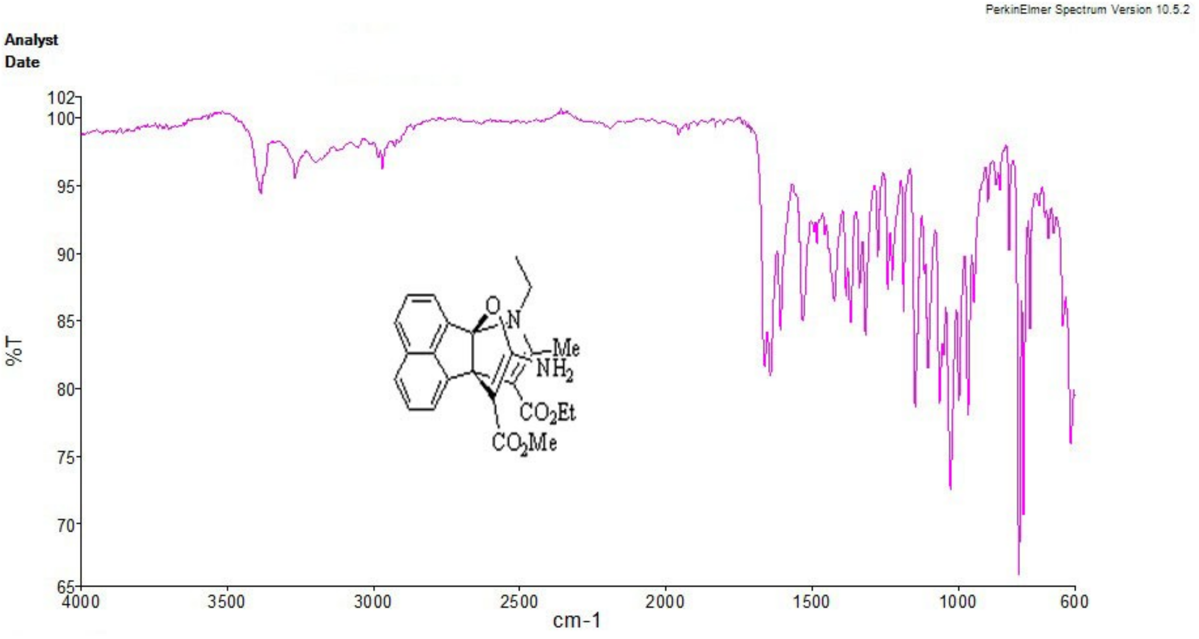

PerkinElmer Spectrum Version 10.5.2
Analyst
Date
102
100
95
90
85
80
75
70
65
%T
4000
3500
3000
2500
2000
1500
1000
600
cm-1
O
N
Me
NH_2
CO_2Et
CO_2Me
